# Flavin-containing siderophore-interacting protein of *Shewanella putrefaciens* DSM 9451 reveals common structural and functional aspects of ferric–siderophore reduction

**DOI:** 10.1007/s00775-025-02106-z

**Published:** 2025-03-13

**Authors:** Inês B. Trindade, Bruno M. Fonseca, Teresa Catarino, Pedro M. Matias, Elin Moe, Ricardo O. Louro

**Affiliations:** 1 Avenida da República (EAN), Instituto de Tecnologia Química e Biológica António Xavier da Universidade Nova de Lisboa, 2780-157 Oeiras, Portugal; 2 Present Address: Division of Biology and Biological Engineering, California Institute of Technology, Pasadena, CA 91125 USA; 3 Departamento de Química, Faculdade de Ciências e Tecnologia, Universidade Nova de Lisboa, 2829-516 Caparica, Portugal; 4 iBET-Instituto de Biologia Experimental e Tecnológica, Apartado 12, 2780-901 Oeiras, Portugal

**Keywords:** Ferric–siderophore reduction, Siderophore-interacting proteins, Siderophore, Iron uptake, *Shewanella*, Flavoprotein, Iron metabolism

## Abstract

*Shewanella* are bacteria widespread in marine and brackish water environments and emergent opportunistic pathogens. Their environmental versatility depends on the ability to produce numerous iron-rich proteins, mainly multiheme *c*-type cytochromes. Although iron plays a vital role in the versatility of *Shewanella* species, very few studies exist regarding the strategies by which these bacteria scavenge iron from the environment. Siderophore-mediated iron transport is a commonly employed strategy for iron acquisition, and it was identified among *Shewanella* spp. over two decades ago. *Shewanella* species produce hydroxamate-type siderophores and iron removal from these compounds can occur in the cytoplasm via Fe(III)–siderophore reduction mediated by siderophore-interacting proteins (SIPs). The genome of *Shewanella putrefaciens* DSM 9451 isolated from an infected child contains representatives of the two different families of SIPs: the flavin-containing siderophore reductase (*Sb*SIP) and the iron–sulfur cluster-containing ferric–siderophore reductase (*Sb*FSR). Here, we report their expression, purification, and further biochemical characterization of *Sb*SIP. The structural and functional characterization of *Sb*SIP and comparison with the homologous SIP from *Shewanella frigidimarina* (*Sf*SIP) revealed similarities between these proteins including a common binding pocket for NADH, NADPH, and siderophore substrates plus a pronounced redox-Bohr effect that ensures coupled transfer of electrons and protons in the physiological pH range. These mechanistic aspects open the door for further investigations on developing drugs that interfere with the iron metabolism of these bacteria and thereby prevent their spread.

**Graphical abstract:**

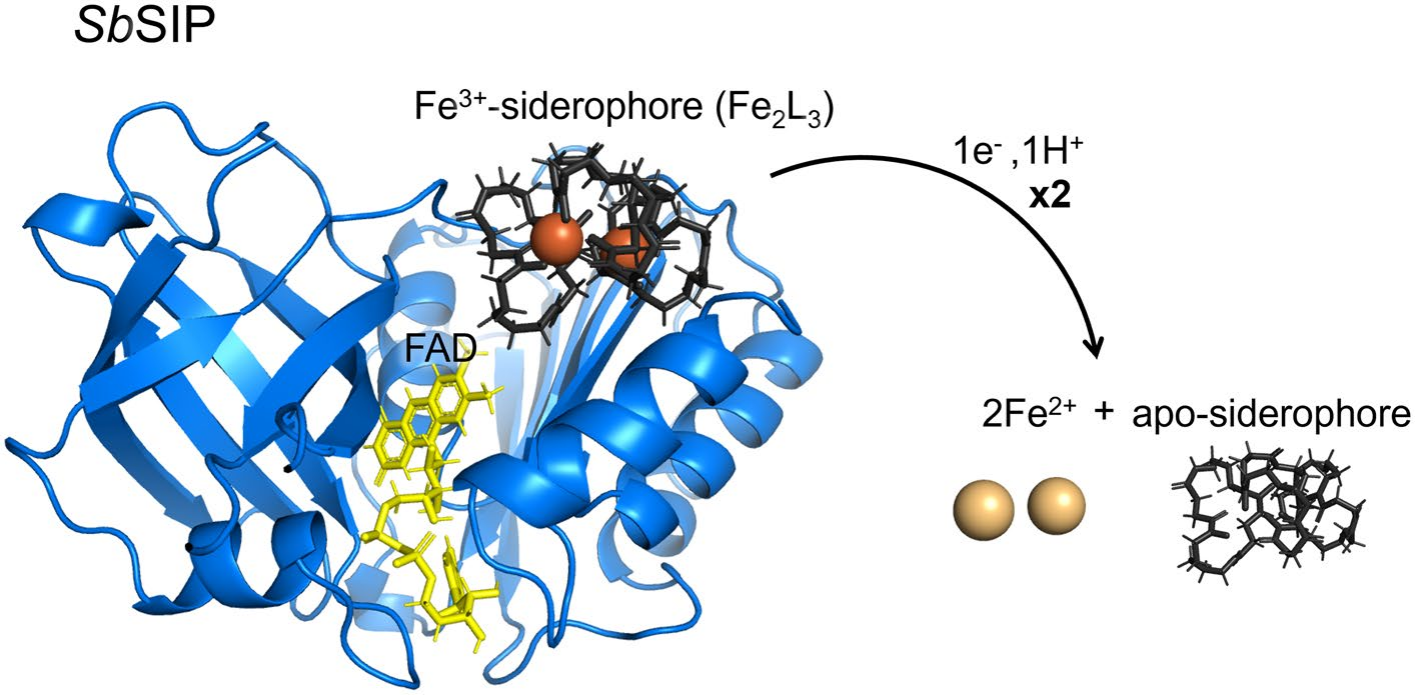

**Supplementary Information:**

The online version contains supplementary material available at 10.1007/s00775-025-02106-z.

## Introduction

Iron is a key element for life, participating in various biological processes by mediating fundamental redox reactions via the incorporation into numerous proteins [1–3]. Iron, though abundant, is not easily bioavailable, as it precipitates into ferric minerals in oxygen-rich environments [4, 5]. To overcome iron shortage, microorganisms produce and release siderophores into the environment. Siderophores are secondary metabolites with a high affinity for ferric iron, Fe(III), which solubilize and scavenge iron for intracellular uptake [6–8]. The main steps of the siderophore pathway include intracellular synthesis, extracellular release, ferric-iron complexation, cellular uptake, and iron release [9, 10]. Once inside the cell, given the high affinities for Fe(III) and the very negative reduction potentials of Fe(III)–siderophores, iron release from these compounds can occur via a single mechanism or through a combination of different mechanisms: the hydrolysis of the Fe(III)–siderophore, redox-shift hypothesis, and/or reduction of the ferric iron within Fe(III)–siderophore complexes. The hydrolysis of the Fe(III)–siderophore is mediated by esterases and it was observed for various siderophores including bacillibactin (by BesA esterase), enterobactin (by esterases Fes and PfeE), and salmochelin (by esterase IroD and IroE). Hydrolysis alone is often not enough to make iron accessible. Therefore, hydrolysis is frequently followed by a reduction step, as observed in the release of iron from Fe(III)–enterobactin in *E. coli* and *P. aeruginosa *[11, 12]. The redox-shift hypothesis integrates the various ways of increasing the reduction potential of siderophores to ease iron release [10, 13–15]. This includes siderophore modification (e.g., acetylation), proton-assisted dissociation of the siderophore complex, media lipophilicity, coupled Fe(II) chelation, and ternary complex association. At neutral pH and in the absence of ester bonds, the recurring mechanism is the release of iron from Fe(III)–siderophores via reduction of the Fe(III) iron. Upon reduction of Fe(III) to Fe(II), the stability of the complex is dramatically decreased given the lower affinity by 20 log units of siderophore ligands to Fe(II) [13, 16, 17]. This positively impacts the kinetics of ligand exchange allowing time- and site-specific delivery of the metal. Reduction of iron is proposed to occur via small molecule reducing agents or by a superfamily of assimilatory Fe(III)-reductases. Within these, two families exist of cytoplasmic Fe(III)-reductases: the siderophore-interacting protein (SIP) family or the ferric–siderophore reductase family (FSR). SIPs contain stably attached flavins and use NADH and/or NADPH as reducing agents. These are widely conserved among bacteria most frequently in proteobacteria (64%) and in actinobacteria (33%), but also others [18]. The FSR family has the prototypical example of FhuF from *E. coli* K-12, an atypical 2Fe-2S ferredoxin, that is involved in the iron reduction of coprogen, ferrichrome, and ferrioxamine B. Few studies have explored the function and mechanism of FSRs. As a result, these two enzyme families are considered to have a redundant role: reducing ferric iron complexed with siderophores to its ferrous form, which decreases the complex’s affinity and facilitates iron release [12, 18–21].

The *Shewanella* genus is well known for its respiratory versatility, an ability that is facilitated by an extensive repertoire of iron-containing proteins, specifically, multiheme cytochromes [22, 23]. Because of this, *Shewanellaceae* have higher iron requirements than many well-known bacteria, e.g., approximately fourfold more than *Escherichia coli* [24]. Despite their importance, there is still much to be learned regarding the iron acquisition pathways of this genus. Two decades ago, the iron-sequestering abilities of 51 strains of *Shewanella putrefaciens* isolated from different sources (fish, water, and warm-blooded animals) were assessed, where more than half of the strains produced hydroxamate-type siderophores [25]. It is now becoming increasingly recognized that to thrive in a wide diversity of iron-deficient habitats ranging from the ocean bed to the eukaryotic host, *Shewanella* species adapt metabolically, including through the production of different siderophores and subsequent different proteins for their utilization [26, 27]. Some examples include the production of cyclic dihydroxamate putrebactin by *S. putrefaciens*, and the production of asymmetrical avaroferrin by *S. algae*. Also, different *Shewanella* species have different Fe(III)–siderophore reductases [28]. For instance, *S. frigidimarina* produces only a representative of the SIP family (*Sf*SIP), and S. *oneidensis* only produces a representative of the FSR family (PutB), whereas some species (e.g., *S.algae*) produce representatives of both SIP and FSR families [18, 21, 27, 29–31].

In this work, we investigated siderophore-iron release from *S. putrefaciens* (DSM 9451) also known as *Shewanella* sp. JAB-1 and tentatively classified it as *S. bicestrii* based on its genome sequence [32]. *S. bicestrii* was first identified as part of three extended spectrum β-lactamase (ESBL)-producing bacteria in a bile sample of a 10-year-old child suffering from cholangitis [33].

Here, we report the production of both siderophore-interacting proteins, the flavin-containing (*Sb*SIP) and iron–sulfur cluster-containing ferric–siderophore reductase (*Sb*FSR) from *S. biscestrii*. Given the instability of the latter, we only report the structure and biochemical characterization of *Sb*SIP.

## Materials and methods

### Production of *Sb*SIP and *Sb*FSR

*Sb*FSR and *Sb*SIP expression vectors were designed based on the SLIC (Sequence and Ligation Independent Cloning) method developed by Scholz and co-workers [34]. Primers were designed as described therein to include an HRV 3C protease cleavage site and ccdB counter-selection (3C-LP1 and ccdB-LP2). Gene fragments *sbsip* and *sbfsr* were amplified using KAPA2G Robust PCR kit from a colony of commercially available *S. putrefaciens* DSM 9451 (DSMZ) grown on a Luria–Bertani (LB) agar plate. PCR fragments were then ligated into pCoofy38, a vector containing an N-terminal thioredoxin-his_10_ tag using a Gibson Assembly® Cloning Kit (New England BioLabs). Plasmids were extracted and transformed into competent *E. coli* BL21(DE3) cells for expression. Proteins were expressed by growing expression strains in LB Broth Media (*Sb*SIP) or Terrific Broth Media (*Sb*FSR) supplemented with 50 mg/L kanamycin at 37 °C, 150 rpm, and expression was induced with 1 mM IPTG at an OD_600_ of 0.5–0.7. After approximately 30 h, cells were harvested by centrifugation and frozen at − 80 °C. Cells were later defrosted and resuspended in 20 mM potassium phosphate buffer, pH 7.6, with 300 mM NaCl and a protease inhibitor cocktail (Roche) together with DNase I (Sigma) prior to a three-pass cell disruption at 6.9 MPa using a French press. The lysate was ultracentrifuged at 204,709×*g* for 75 min at 4 °C to remove cell membranes and debris. Proteins were purified from the supernatant using a His-trap affinity column (GE Healthcare) with a stepwise elution method. The fraction containing *Sb*FSR was eluted at 20 mM potassium phosphate, pH 7.6, 300 mM NaCl with 250 mM imidazole, whereas *Sb*SIP was eluted with 150 mM imidazole. Eluted fractions were analyzed by SDS-PAGE using Blue Safe staining (NZYTech) and UV–visible spectroscopy to select fractions containing *Sb*FSR and *Sb*SIP. The fractions of each protein were pooled, the imidazole was removed through dialysis overnight, and proteins were concentrated using an Amicon ® Ultra Centrifugal Filter (Millipore) with a 30 kDa cutoff. The *Sb*FSR yield was very low (4mg/L versus 30mg/L for *Sb*SIP), and fractions precipitated after concentration. Thus, Trx-His_10_ tag cleavage was only performed for *Sb*SIP. *Sb*SIP fractions were incubated with HRV 3C protease overnight at 4 °C with agitation, and the final purified *Sb*SIP was concentrated from the flow-through of a second passage through the His-trap column using an Amicon Ultra Centrifugal Filter (Millipore) with a 30 kDa cutoff. The purity of *Sb*SIP was confirmed by SDS-PAGE using Blue Safe staining (NzyTech), N-terminal sequencing analysis, and UV–visible spectroscopy. An extinction coefficient of free FAD *ε*_450nm_ = 11 300 M^−1^ cm^−1^ was used for quantification purposes [35].

### Crystallization and structure determination of *Sb*SIP

Purified *Sb*SIP (Trx-His10 tag-free) at a concentration of 10 mg/ml was crystallized by the hanging drop vapor diffusion technique using as precipitant a solution containing 1.8 M ammonium sulfate with 0.01 M cobalt(II) chloride hexahydrate and 0.1 M MES, pH 6.5. Drops containing 1 μL protein and 1 μL reservoir were equilibrated against 500 μL reservoirs in a 24-well plate (Hampton Research). Crystals were harvested and soaked in a 2 μL drop of cryo solution (1.8 M ammonium sulfate 0.1 MES pH 6.5 with 30% glycerol) prior to flash-freezing in liquid nitrogen. Diffraction data were collected at 100 K to a resolution of 1.86 Å at the ALBA beamline XALOC (Barcelona, Spain). The images were processed with AutoProc and STARANISO, which make use of XDS and the CCP4 suite for integration and conversion of integrated intensities to structure factors [36–41]. The data processing statistics are listed in Table 1. The structure was solved by molecular replacement using PHASER in the CCP4 suite and a previously determined SIP crystal structure from *S. putrefaciens* (PDB 2GPJ, Joint Center for Structural Genomics) as phasing model. The asymmetric unit cell of the crystal contained two *Sb*SIP protein chains (A and B), each one bound to an FAD moiety, and the model was automatically corrected with BUCCANEER/REFMAC in the CCP4 suite [42, 43].

**Table 1:** Data collection and processing statistics

Beamline	ALBA XALOC
Detector	PILATUS 6 M
Wavelength (Ǻ)	0.97918
Data processing	AutoProc/STARANISO
Space group	*P* 4_3_ 2_1_ 2
Unit cell parameters (Ǻ)	*a* = 82.44, *c* = 250.85
Resolution limits of ellipsoid fitted to resolution cutoff surface (Å)	2.29, 2.29, 1.71
Resolution, spherical limits (Å)	78.32–1.86 (2.03–1.86)
Nr. observations	317,355 (13,920)
Unique reflections	49,068 (2453)
Multiplicity	6.5 (5.7)
Completeness, spherical (%)	66.9 (15.0)
Completeness, ellipsoidal (%)	93.7 (68.2)
R-merge (%)^a^	5.1 (74.6)
R-meas (%)^b^	5.6 (82.0)
CC^1/2^	1 (0.819)
<*I*/*σ*(I)>	19.3 (2.2)
ISa	36.6
Wilson plot B (Å^2^)	44.2
*Z*^c^	2
*V*_m_	3.72
Estimated solvent content (%)	67.0

^a^*R*-merge = merging *R*-factor, (*Σ*_*hkl*_ *Σ*_*i*_ |*I*_*i*_(*hkl*) − < *I*(*hkl*) >|)/(* Σ*_*hkl*_ *Σ*_*i*_ *I*(*hkl*)) ×100%

^b^*R*-meas = redundancy independent *R*-factor, *Σ*_*h*_ [*N*_*hkl*_/(*N*_*hk*l_ − 1)]^1/2^ *Σ*_*i*_|*I*_*i*_(*hkl*) − < *I*(*hkl*)>|/*Σ*_*hkl*_ *Σ*_*i*_ *I*_*i*_(*hkl*) × 100%, where *I* is the observed intensity, <*I*> is the average intensity of multiple observations from symmetry-related reflections, and *N*_*hkl*_ is their multiplicity [40]

^c^Nr. monomers in the asymmetric unit according to Matthews coefficient [41]

After an initial refinement using REFMAC5 in the CCP4 suite, structure refinement was continued using PHENIX [44]. Hydrogen atoms were included in calculated positions with the PHENIX READYSET tool, and isotropic atomic displacement parameters (ADPs) were refined for all non-hydrogen atoms. Throughout the refinement, the model was periodically checked and corrected with COOT against *σ*_A_-weighted 2|*F*_o_| − |*F*_c_| and |*F*_o_| − |*F*_c_| electron-density maps. Solvent molecules were added automatically by the ArpWarp solvent protocol via the CCP4 suite and validated by inspection of electron-density maps in COOT [45, 46]. In the final refinement cycles, a TLS rigid body refinement of the ADPs was carried out, considering seven and six rigid body groups for *Sb*SIP chains A and B, respectively, determined with the PHENIX FIND_TLS_GROUPS tool from a previous refinement with isotropic ADPs. The final values of R and R-free were 0.168 and 0.200, respectively, with a maximum likelihood estimate of the overall coordinate error of 0.17 Å [47]. The refinement statistics are presented in Table 1. The model stereochemical quality was analyzed with MOLPROBITY and there were no outliers in the Ramachandran φ, ϕ plot [48]. The coordinates and structure factors have been submitted to the Worldwide Protein Data Bank with accession code 8C4L. Images were produced using PyMOL [49].

### Protein film voltammetry (PFV) of *Sb*SIP

PFV experiments of *Sb*SIP were performed at 25 °C using a three-electrode electrochemical cell configuration with a PGE (pyrolytic graphite edge) electrode, a graphite rod (counter electrode), and an Ag/AgCl 3 M KCl (reference electrode) inside a Coy anaerobic glovebox chamber using a CHI electrochemical analyzer (CHI instruments). The electrode was cleaned and freshly polished before every experiment. The polishing routine consisted of a 10 min nitric acid incubation at room temperature followed by 10 min of hand polishing with a 1.0 μM alumina aqueous slurry. The electrode was thoroughly rinsed with water and left to dry and then *Sb*SIP was immobilized by pipetting 7 μL of a 250 μM solution of *Sb*SIP in 20 mM potassium phosphate buffer at pH 7.6 with 100 mM KCl. Once fully dried, the electrode was rinsed to remove protein excess and immersed in potassium phosphate buffer at different pH values. Experiments were performed at different scan rates and then the buffer was collected, and the pH was measured for confirmation. QSoas was used to subtract the capacitive current and extract the reduction potentials. Potentials are reported in mV versus the standard hydrogen electrode (SHE) by the addition of 210 mV to those measured [50].

### ^31^P NMR: NAD(P)H binding experiments

NADH, NADPH, and *Sb*SIP were prepared in 20 mM Tris–HCl buffer at pH 8 with 100 mM KCl containing 10% of ^2^H_2_O (99.9 atom %). Using the standard Bruker pulse program “zgdc,” one-dimensional proton-decoupled ^31^P spectra were acquired with 2048 scans, d1 of 1.3 s at 25 °C on a Bruker Avance II 500 MHz equipped with a SEX probe for ^31^P detection. Samples of 100 µM or 57 µM of NADH and NADPH, respectively, were titrated against increasing concentrations of *Sb*SIP. Collected spectra were visualized and analyzed using TopSpin 3.6 (Bruker). For NADH binding, the concentration of free and bound species was determined from the relative intensity of each peak, and the dissociation constant (*K*_d_) was calculated using the equation previously described [21]. For NADPH binding, the chemical shift perturbations (Δ*δ*) of the NMR signals from NADPH that resulted from the complex formation with *Sb*SIP in the fast exchange regime were plotted against the molar ratio (*R*) of [*Sb*SIP]/[NADPH]. Results were fitted and the dissociation constant and the respective uncertainty (*K*_d_) was determined as described by Fonseca et al. [51].

### Kinetic experiments

The kinetic experiments were performed with HI-TECH Scientific Stopped-flow equipment (SF-61DX2) installed inside an anaerobic glove box (Mbraun MB150-GI). The temperature of the drive syringes and mixing chamber was maintained at 25 °C using a water bath. Sample solutions were prepared with 20 mM potassium phosphate buffer, pH 7, with 100 mM KCl and in the presence of an O_2_ scavenging system (10 mM glucose, 375 nM glucose oxidase, and 750 nM catalase). The time course of the reactions was monitored using a photodiode array. Solutions were prepared inside the anaerobic chamber with degassed water and all experiments were performed in triplicate. Data were analyzed with Kinetics Studio version 2.32 (TgK Scientific).

Reduction of both *Sb*SIP and *Sf*SIP with NADH and NADPH was performed by mixing 1 mM of these compounds with 20 μM of *Sb*SIP or *Sf*SIP. The reduction with sodium dithionite was performed by mixing 3 mM of this compound with 20 μM *Sb*SIP.

Fe(III)–siderophores putrebactin and bisucaberin were kindly provided by Prof. Masaki Fujita. Reduction of these by reduced *Sb*SIP (*Sb*SIP_semi_) was performed after reducing *Sb*SIP with sodium dithionite. The latter was achieved by using excess sodium dithionite which was then removed through buffer exchange in a HiTrap® Desalting Column (GE Healthcare). Ferric–siderophore reduction experiments were then performed using 20 μM *Sb*SIP_semi_ against 100 μM of ferric–siderophore in the stopped-flow apparatus. The reduction rate constants were obtained from the fitting of the kinetic traces at 600 nm. Catalytic experiments with NADH or NADPH and ferric–siderophores were attempted. However, spectra overlap prevented an unambiguous interpretation of the results, and thus these data were excluded from this manuscript. Ferrozine assays were also attempted. However, in the presence of the oxygen-scavenging system, absorption changes were observed in the absence of *Sb*SIP (control). Ferrozine is a very strong ferrous iron chelator and in the absence of oxygen, the shift in chemical equilibrium is sufficient for Fe(III)–siderophore reduction to take place at detectable rates without enzymatic mediation [52].

### Docking NADH, NADPH and Fe(III)–siderophores

The ligand docking calculations were executed through the High Ambiguity Driven Docking 2.4 (HADDOCK 2.4) webserver, using the 8C4L PDB structure of *Sb*SIP and the 6GEH PDB structure of *Sf*SIP and incorporating the substrates Fe(III)–bisucaberin, Fe(III)–alcaligin, NADH, and NADPH, in separate runs [53, 54]. In each run, 10,000 rigid-body solutions were generated via energy minimization. Subsequently, the 400 structures with the lowest ambiguous interaction restraints (AIRs) underwent semi-flexible simulated annealing in torsion angle space, followed by a final refinement in explicit water. The resulting water-refined structures were clustered using a 1.5 Å backbone root mean square deviation (RMSD) cutoff and ranked based on their HADDOCK score. Structures with the lowest HADDOCK scores were chosen for further analysis and visualized using PyMOL [55].

## Results and discussion

### Production of the Fe(III)–siderophore reductase *Sb*SIP

**Fig. 1:**
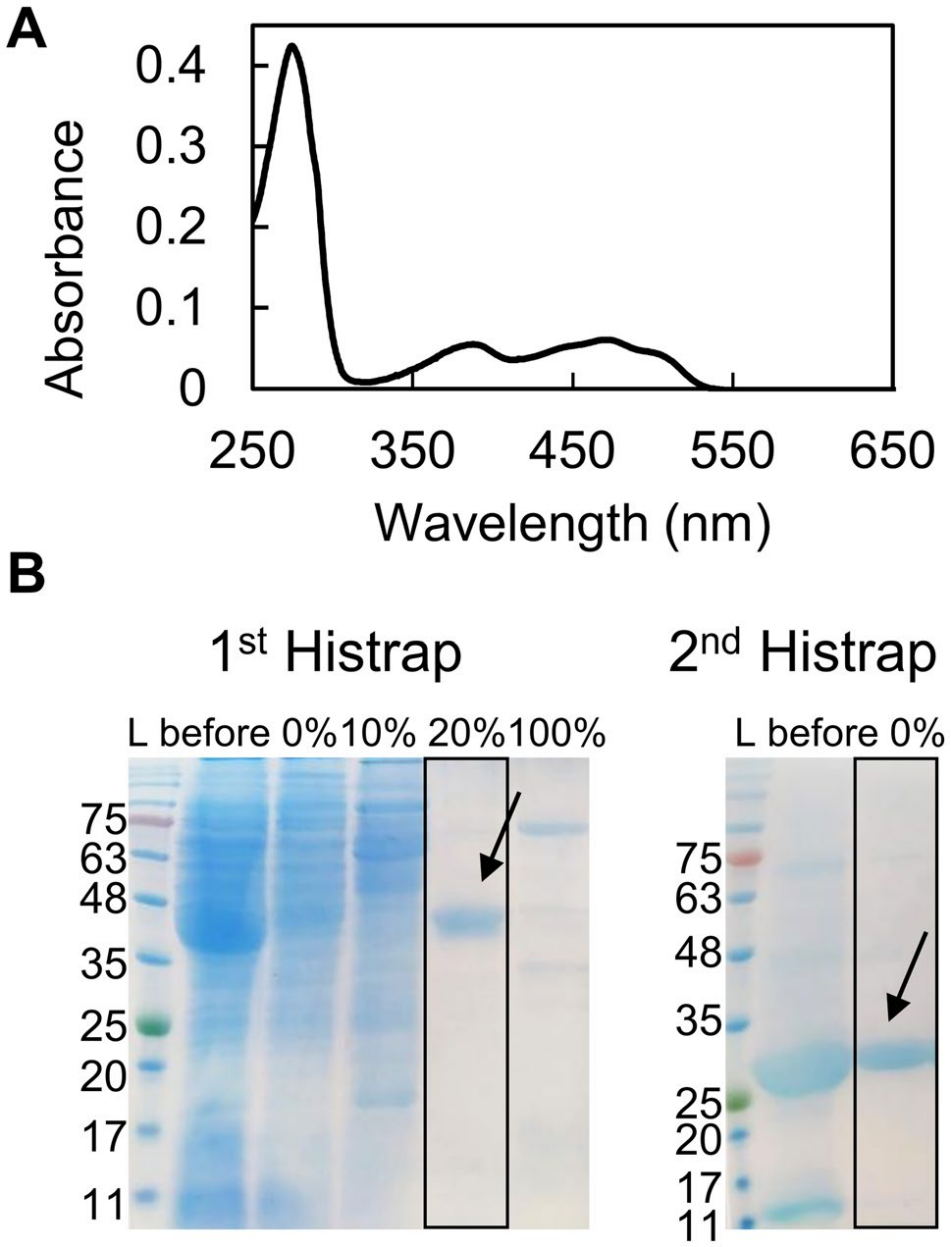
Production of *Sb*SIP. **A** UV–visible profile of pure oxidized *Sb*SIP showing the typical spectrum with maxima close to 387 nm and 471 nm. **B** SDS-PAGE gels of purification steps of *Sb*SIP before (1st HisTrap purification) and after HRV 3C incubation step (2nd HisTrap purification). Percentages represent the amount of imidazole used out of a 500 mM stock solution

*Sb*SIP was heterologously expressed in *E. coli* and purified to apparent purity (> 95%). It migrated as a single band at approximately 30 kDa (42 kDa before incubation with the HRV 3C protease to remove the thioredoxin-His_10_ tag) on a 15% SDS–PAGE gel, as expected from theoretical calculations (Fig. 1). The purified protein appeared yellow and the UV–visible spectrum showed the typical spectral features of an oxidized flavoprotein in the UV–visible region (Fig. 1A), with absorption peaks at 387 nm and 471 nm and distinct shoulders located at 445 nm and 502 nm. *Sb*FSR was not purified to complete purity (Fig. S1) and, given that the sample yield was very low and precipitated shortly after the first purification step, we did not proceed with its biochemical characterization. Regardless, the fractions containing *Sb*FSR were reddish brown, and the UV–visible spectra showed the typical spectral features of an oxidized 2Fe–2S protein, with maximum absorption peaks at 340 nm and 451 nm (Fig. S1). The sequence of SbFSR shows identities of 35% to FhuF (*Escherichia coli* K-12) and 22% to FchR (*Alkalihalophilus pseudofirmus*). It has an identical cluster binding motif sequence (C–C-x_10_–C-x_2_–C) and AlphaFold2 predicts an overall folding conservation when compared to the archetypical FhuF from *E. coli* [56, 57]. However, *Sb*FSR contains three extra α-helices and a longer N-terminal loop (Fig. S2).

### The structure of *Sb*SIP emphasizes putative sites for substrate specificity

**Fig. 2:**
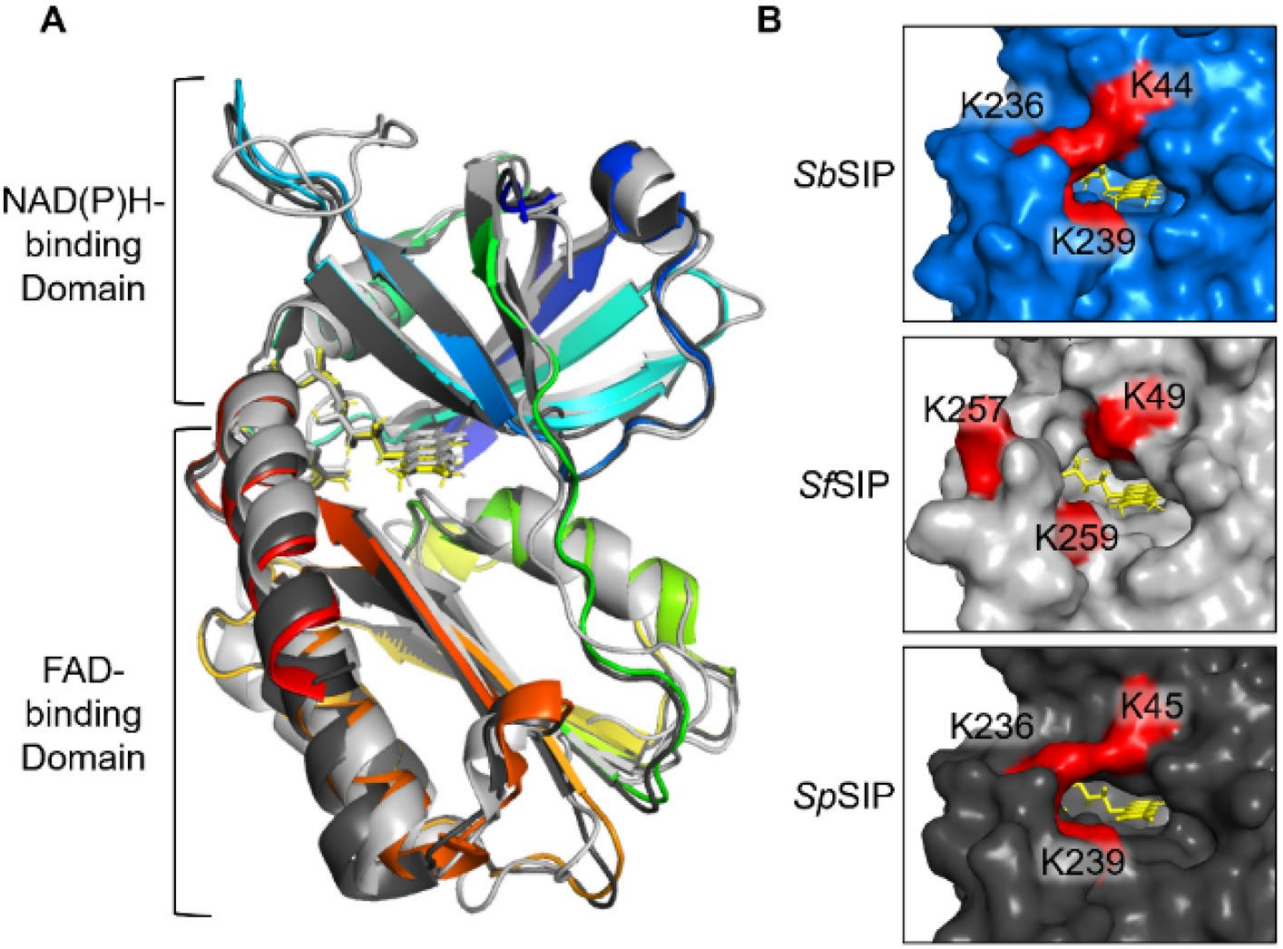
Structural characterization of *Sb*SIP. **A** Structure of *Sb*SIP (blue to red from the N to the C terminal, PDB 8C4L) versus *Sf*SIP (light gray, PDB 6GEH), and *Sp*SIP (dark gray, PDB 2GPJ) aligned with PyMOL. **B** Molecular surfaces of *Sb*SIP, *Sf*SIP, and *Sp*SIP highlighting in red the lysine triad pockets [55]

The structure of *Sb*SIP was determined at 1.9 Å resolution (Fig. 2, Table 2). The structure revealed two domains as previously described for other SIPs: a N-terminal or NAD(P)H binding domain and a C-terminal or FAD binding domain. The NAD(P)H binding domain consists of the typical β1–α1–β2 Rossmann fold architecture composed of a manifold of β-antiparallel-strands (β1–β6) and two short α-helices (α1 and ƞ1). The FAD binding domain is composed of five antiparallel β-strands (β7–β11) with two short α-helices (α2 and α3) connected by long loops. A DALI search (http://ekhidna2.biocenter.helsinki.fi/dali/) for structures similar to that of *SbSIP* reveals 4915 similar non-unique structures (i.e., all chains are listed for crystal structures with more than one chain in the asymmetric unit) with a *Z*-score above 2.0, while a similar search using PDBeFold (https://www.ebi.ac.uk/msd-srv/ssm/) yields 115 non-unique matches with a Q-score above 0.35. While these results highlight the prevalence in nature of the domain folds found in *Sb*SIP, the top hits in both searches correspond to seven related unique siderophore-interacting protein structures (Table S1) [58, 59].

**Table 2:** Final refinement statistics of *Sb*SIP

Resolution limits (Å)	62.71–1.86 (1.90–1.86)
*R*-factor (%)^a^	16.9 (20.0)
nr. reflections	46,650 (155)
Free *R*-factor (%)^b^	17.4 (27.6)
nr. Reflections	2412 (8)
Overall coordinate error estimate (Å) ^c^	0.17
Model composition	
Non-hydrogen protein atoms	3826
Solvent molecules	323
FAD ligand	106
Model r.m.s. deviations from ideality	
Bond lengths (Å)	0.012
Bond angles (°)	1.15
Chiral centers (Å^3^)	0.063
Planar groups (Å)	0.012
Model completeness and validation	
Regions omitted	1–3 and 247–251 in chain A 1–5 and 249–251 in chain B
Mean *B* values (Å^2^)^d^	
Protein	39.0
solvent molecules	39.4
FAD ligand	28.6
Ramachandran plot statistics. Residues in:	
Most favored regions (%)	96.3
Allowed regions (%)	3.7
Disallowed regions (%)	0.0
Rotamer outliers (%)	1.2
C^β^ outliers	0.0
Clash score	2.05
MolProbity score	1.29

^a^*R*-factor = *Σ*_*hkl*_ ||*F*_o_| − |*F*_c_||/*Σ*_*hkl*_ |*F*_o_|, where |*F*_o_| and |*F*_c_| are the observed and calculated structure factor amplitudes, respectively

^b^Free *R*-factor is the cross-validation *R*-factor computed from a randomly chosen subset of 5% of the total number of reflections, which were not used during the refinement

^c^Maximum-likelihood estimate with PHENIX

^d^Calculated from the equivalent isotropic *B* values

**Fig. 3:**
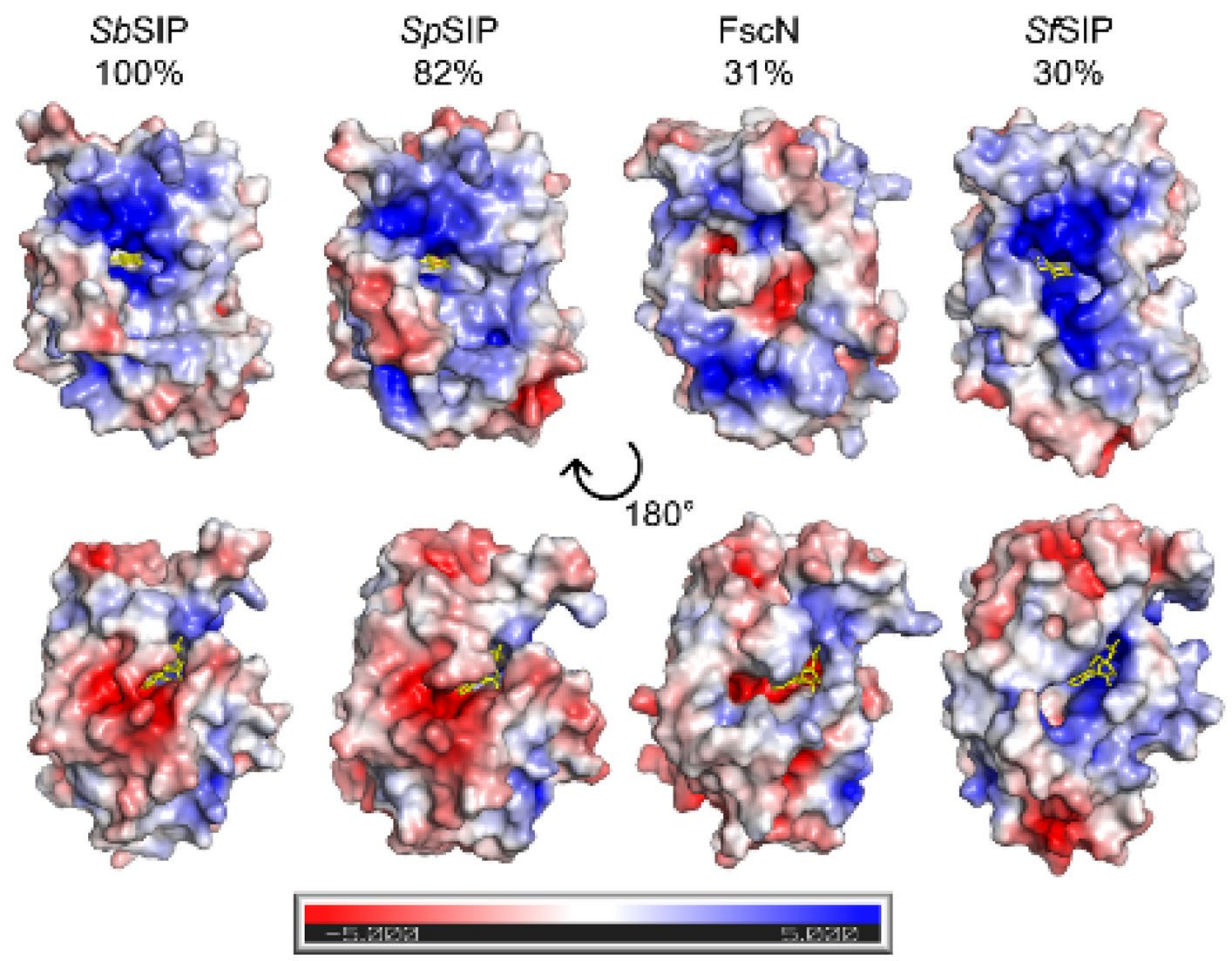
Electrostatic surface potential (− 5 to + 5 kT/e) calculated for various SIPs: *S. bicestrii* (*Sb*SIP, PDB 8C4L), *S. putrefaciens* (*Sp*SIP, PDB 2GPJ), *T. fusca* (FscN, PDB 4YHB) and *S. frigidimarina* (*Sf*SIP, PDB 6GEH). The surfaces were calculated using the APBS plugin in PyMOL [60], and are shown in the same orientation. The top row highlights the access through the isoalloxazine ring (represented as yellow sticks), and the bottom row shows all surfaces after a clockwise 180° rotation about a vertical axis. Sequence identity with *Sb*SIP is indicated above each protein

The FAD isoalloxazine ring shows a planar conformation (Fig. S3) and is stabilized through aromatic stacking interactions with Tyr-60 and Tyr-225, and hydrogen bonds with residues Thr-61 (backbone O and N), Tyr-59 (side chain OH), Val-75 (backbone N), and Asp-73 (backbone O). The negatively charged phosphate groups of FAD are also stabilized through hydrogen bonds with Thr-60 (backbone N), Glu-232 (backbone N), His-77 (side chain Nε2), Gly-81 (backbone N), Ser-84 (Side chain OH), and Ala-83 (backbone N). The triad of basic amino acid residues (Lys-45, Lys-236, and Lys-239 reported in SIP from *S. putrefaciens, Sp*SIP) and proposed to form the Fe(III)–siderophore binding pocket is well conserved (Lys-44, Lys-236, and Lys-239 in *Sb*SIP). As previously observed for *Sp*SIP, Lys-44 forms a ridge with Lys-236, making the access to the FAD cofactor smaller when compared to *Sf*SIP (Fig. 2B). Sequence alignment (Fig.S4) with previously characterized SIPs shows 82% identity with *Sp*SIP (PDB code 2GPJ), 31% with FscN from *Thermobifida fusca* (PDB code 4YHB), 30% with *Sf*SIP (PDB code 6GEH), and 28% with YqjH from *E. coli* (no structure available). Indeed, when superimposing the structures of *Sb*SIP and *Sf*SIP, very few differences are observed in the overall fold. However, the electrostatic surface potential of these two proteins is remarkably different (Fig. 3). Both *Sb*SIP and *Sf*SIP contain very positively charged pockets that provide access to the FAD cofactor through the isoalloxazine ring. However, the pocket of *Sb*SIP is substantially smaller when compared to *Sf*SIP. It is likely that these differences in the access to the FAD cofactor provide selection factors to discriminate redox partners, including Fe(III)–siderophores and the electron donors NADH and NADPH.

### *Sb*SIP binds preferentially to NADH

**Fig. 4:**
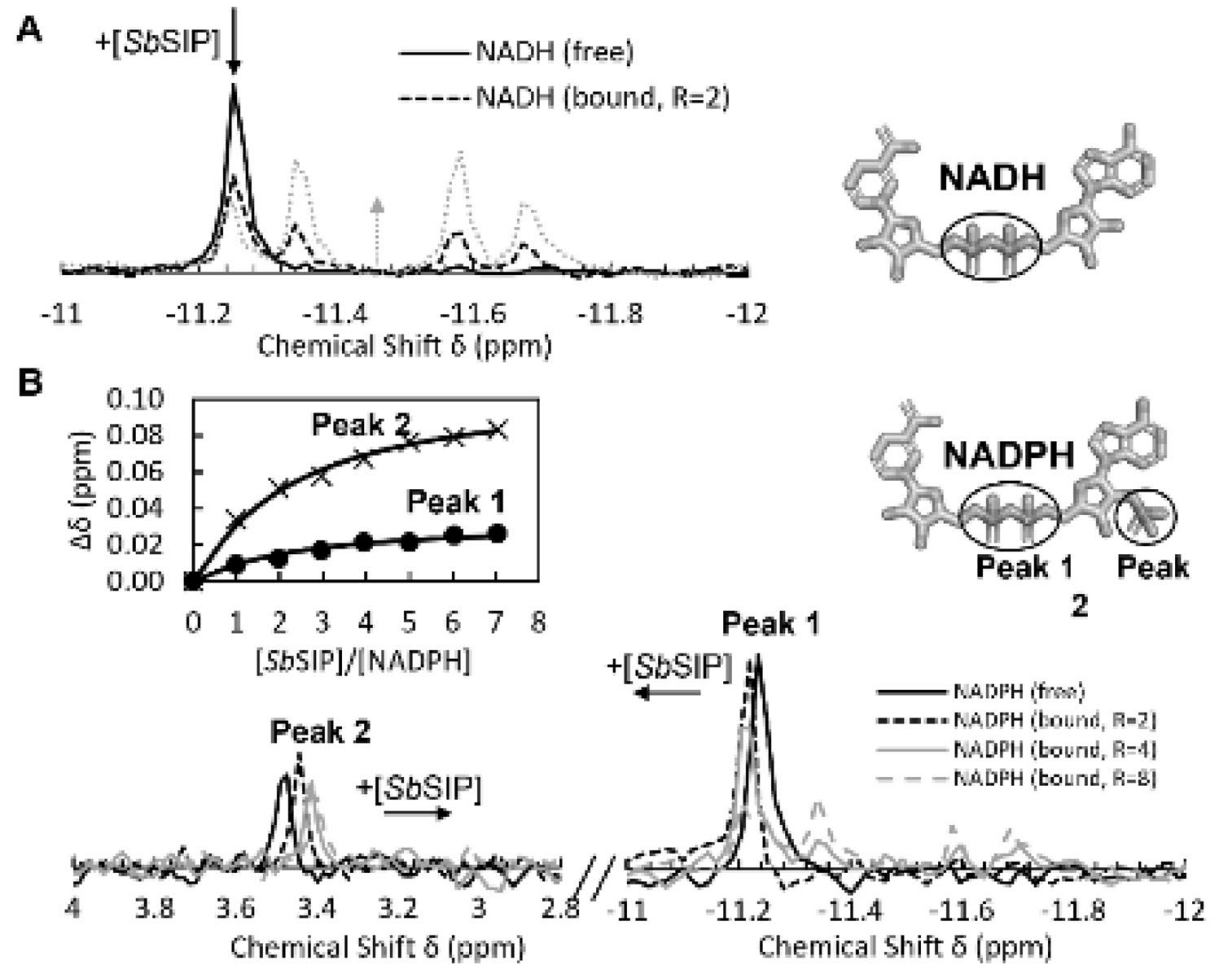
^31^P NMR binding experiments of NADH and NADPH versus *Sb*SIP. **A** Proton-decoupled spectra corresponding to 100 μM of NADH and changes with increasing amounts of *Sb*SIP. R represents the ratio of [*Sb*SIP]/[NADH]. **B** Proton-decoupled spectra corresponding to 57 μM of NADPH and changes with increasing amounts of *Sb*SIP. Inset shows binding curve monitoring the chemical shift perturbation. Peak 1 corresponds to the pyrophosphate phosphorous atoms, whereas peak 2 corresponds to the 2′ phosphate group. *R* represents the ratio of [*Sb*SIP]/[NADPH]

**Fig. 5:**
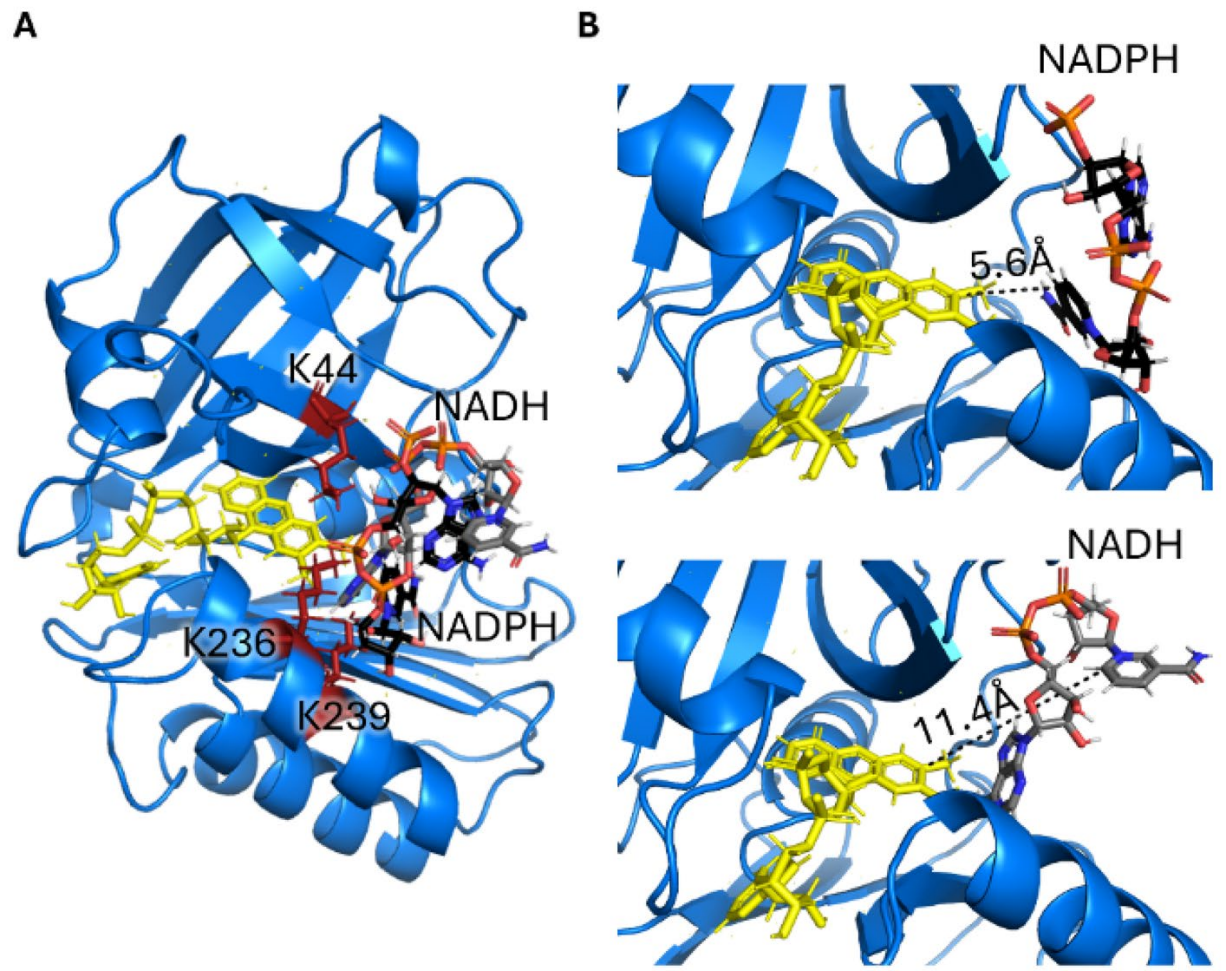
Representation of the binding conformations of NADPH and NADH to *Sb*SIP calculated using Haddock. **A** The binding of NADH (gray) and NADPH (black) occurs in the same region of the isoalloxazine ring of the FAD cofactor (yellow) which is surrounded by the lysine triad (red). **B** zoom of NADPH (top) and NADH (bottom) binding pockets highlighting the shortest distances found between the FAD cofactor and NADPH and NADH, respectively

The high homology with previously characterized SIPs and the presence of a NAD(P)H binding domain in the structure of *Sb*SIP suggests that NADH or NADPH can serve as redox partners. To confirm this hypothesis, we took advantage of the phosphate groups in NADH and NADPH and used ^31^P NMR spectroscopy. Upon mixing *Sb*SIP against NADH and NADPH, significant chemical shift changes (Fig. 4A, B) were observed in the ^31^P spectra of these compounds confirming that *Sb*SIP binds NADH and NADPH as observed for previously characterized *Sf*SIP [21]. The binding of NADH to *Sb*SIP led to changes in the ^31^P signals of the pyrophosphate in the slow-exchange regime in the NMR timescale, giving rise to the coexistence of resonances for the free and bound states with intensities that change with the ratio of *Sb*SIP vs NADH. As for NADPH binding to *Sb*SIP, the pyrophosphate signals and the 2′ phosphoros signals displayed gradual changes in position with the change in the ratio of SIP vs NADPH. This shows that binding occurs in the fast-exchange regime in the NMR time scale. Fitting of the data shows nearly one-order-of-magnitude difference between the dissociation constants for NADH and NADPH, with values of 17 ± 5 µM and 107 ± 2 µM, respectively. Both dissociation constants are consistent with transient interactions, although there is a clear preference of *Sb*SIP for NADH. A hint for the molecular reasons that underpin this preference comes from the observation that the 2′ phosphate signal of NADPH is perturbed upon binding to *Sb*SIP, in contrast to what was observed for binding of NADPH to *Sf*SIP [21]. This observation prompted us to perform molecular simulations of the binding of NADH and NADPH to *Sb*SIP and *Sf*SIP (Figs. 5, S5). These simulations indicate that NADH and NADPH bind *Sb*SIP and *Sf*SIP using the same pocket. This pocket has a positively charged electrostatic surface and provides the best access to the isoalloxazine ring of the FAD cofactor (Fig. S5 and Fig. S6). In the case of the binding of NADPH to S*b*SIP, the 2′ phosphate group of NADPH is facing the protein, in agreement with the observed perturbation of its ^31^P NMR signal. In the case of S*f*SIP the 2′ phosphate group of NADPH faces the solvent. Therefore, these results allow us to conclude that the lower affinity of *Sb*SIP for NADPH is a consequence of steric clashing.

Overall, these data support the notion that the smaller access pocket to the FAD cofactor in *Sb*SIP provides a selection filter against NADPH. As a consequence, the dissociation constant of NADPH is raised while keeping that of NADH similar to what was found for *Sf*SIP. *Sf*SIP does not discriminate between the two electron donors that have dissociation constants close to 20 μM [21]. Despite this difference in affinity, the docked configuration of both NADH and NADPH show distances to the FAD isoalloxazine ring that are compatible with biologically relevant electron transfer rates (Fig. 5).

### SbSIP is reduced to the semiquinone state by both NADH and NADPH at similar rates

**Fig. 6:**
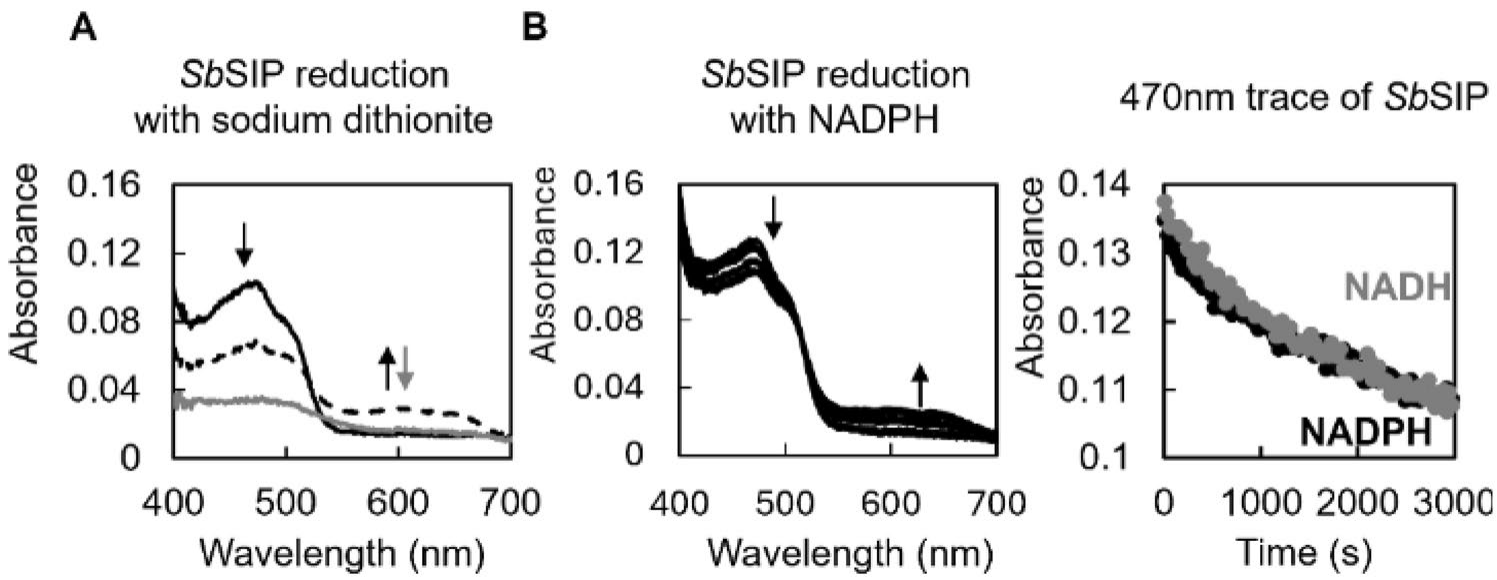
Reduction of *Sb*SIP with various electron donors. **A** Spectral changes observed after mixing *Sb*SIP with sodium dithionite. The black line reports the spectrum of fully oxidized *Sb*SIP, which changes to a state with a significant amount of semiquinone population reported in the dashed line and then to the spectrum of the fully reduced state in gray. Black arrows indicate the decrease at 470 nm and increase at 600 nm occurring upon transition from fully oxidized to the semiquinone state. Gray arrow indicates the decrease in absorbance at 470 nm and 600 nm upon transition from the semiquinone to the hydroquinone state, represented by the continuous gray line. **B** Spectral changes observed after mixing *Sb*SIP with NADH and NADPH and respective kinetic traces at 470 nm. Data for NADH is reported in gray and for NADPH in black. Arrows indicate the direction of spectral changes upon reduction of the oxidized *Sb*SIP

Upon mixing *Sb*SIP with excess amounts of sodium dithionite, absorption spectral changes were characterized by a decrease at 470 nm and an increase at 600 nm, followed by a decrease at both 470 nm and 600 nm. These changes are consistent with the sequential reduction of the oxidized protein to the semiquinone state, followed by reduction to the hydroquinone state (Fig. 6A).

In the presence of the oxygen-scavenging system, it was also possible to observe the partial reduction of *Sb*SIP with NADH and NADPH (Fig. 6). Upon mixing excess amounts of NADH or NADPH with *Sb*SIP, absorption spectral changes were characterized by a decrease at 470 nm and an increase at 600 nm (Fig. 6 and Fig. S7). These changes are consistent with the transition of *Sb*SIP from the oxidized to the semiquinone state. Full reduction into the hydroquinone state was not observed. Despite the differences in dissociation constant found in the ^31^P NMR binding experiments, no significant differences were found in the reduction rate of *Sb*SIP with NADH (0.0003 ± 0.0001 s^−1^) and NADPH (0.0006 ± 0.0005 s^−1^). This indicates that electron transfer is the likely rate-limiting step for *Sb*SIP reduction by NADH and NADPH.

### *Sb*SIP does not perform hydride transfer and displays redox-Bohr effect

**Fig. 7:**
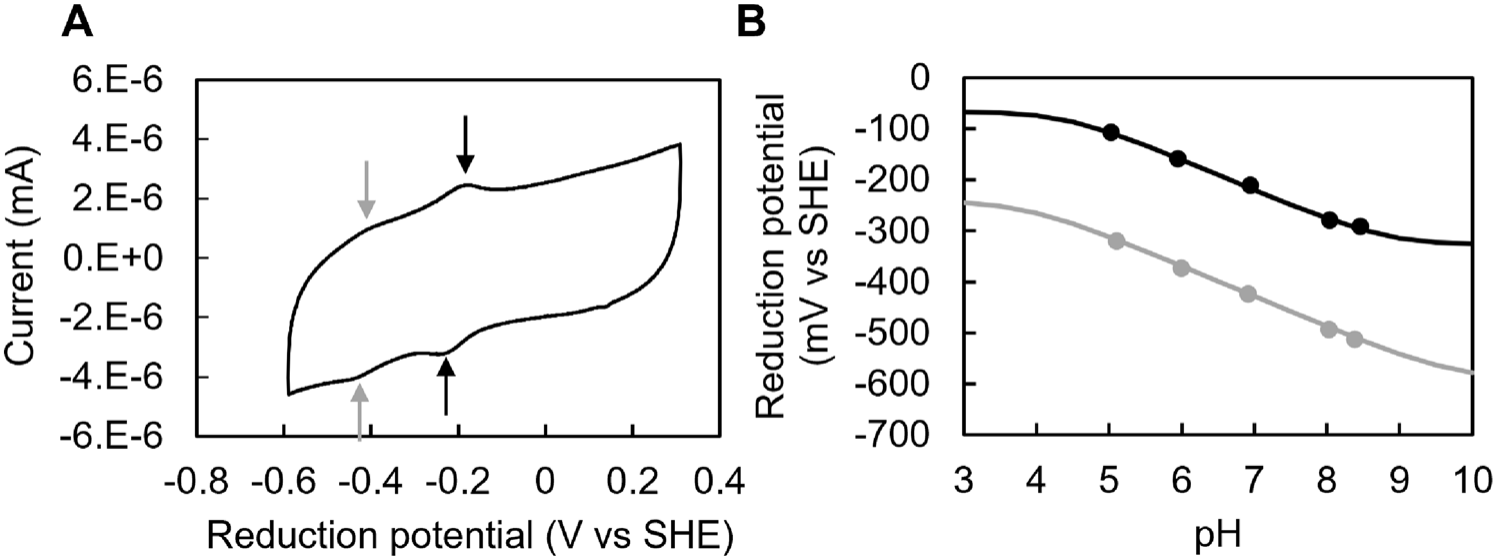
Protein film voltammetry experiments with *Sb*SIP: **A** representative voltammogram of *Sb*SIP, pH 7, at a scan rate of 100 mV s^−1^; gray and black arrows indicate the position of the low and high potential voltammetric signals, respectively, in the anodic and cathodic branch of the voltammogram. **B** pH dependence of reduction potentials of *Sb*SIP with solid line representing the respective simulations, for the low potential signal (gray) and the high potential signal (black)

To determine the redox potentials of *Sb*SIP, we performed protein film voltammetry experiments which showed two well-defined voltammetric signals separated by approximately 200 mV and with a linewidth corresponding to a *n* = 1 electron transfer process (Fig. 7A). These electrochemical properties show that *Sb*SIP does not perform hydride transfer and are significantly different from previously characterized *Sf*SIP where only one voltammetric signal could be observed under similar experimental conditions [21]. The signal at less negative potential corresponds to the transition between the oxidized and the semiquinone state, and the more negative signal corresponds to the transition from the semiquinone to the hydroquinone state. The appearance of this latter signal that is absent in *Sf*SIP highlights once again the structural and electrostatic differences found in the surface of *Sb*SIP vs *Sf*SIP, which can modulate the access of the FAD cofactor to the electrode surface and its potentials. The two voltammetric signals display pH-dependent midpoint potentials (Table 3) which reveal a redox-Bohr effect extending throughout the physiological pH range, with predicted pK_ox_ lower than 4.5 and pK_red_ higher than 8.5 (Fig. 7B). At pH 7, the potential for the transition between the oxidized and the semiquinone state of *Sb*SIP is more positive than that of NADH and of NADPH in agreement with the observation of incapacity of these electron donors to achieve full reduction of the enzyme.

**Table 3:** pH dependence of the midpoint reduction potentials of *Sb*SIP

pH	E1 (V vs SHE)	E2 (V vs SHE)
5.02	− 0.106 ± 0.001	− 0.315 ± 0.005
5.94	− 0.163 ± 0.004	− 0.368 ± 0.005
6.94	− 0.214 ± 0.004	− 0.422 ± 0.003
8.04	− 0.280 ± 0.001	− 0.492 ± 0.002
8.46	− 0.294 ± 0.002	− 0.510 ± 0.003

Values are averages of two experiments and the respective standard error of the mean (SEM)

Having shown that *Sb*SIP can be reduced to the semiquinone or the hydroquinone state depending on the reducing agent, we set out to confirm that it can perform ferric–siderophore binding and reduction.

### *Sb*SIP binds and reduces hydroxamate siderophores produced by *Shewanella* spp.

**Fig. 8:**
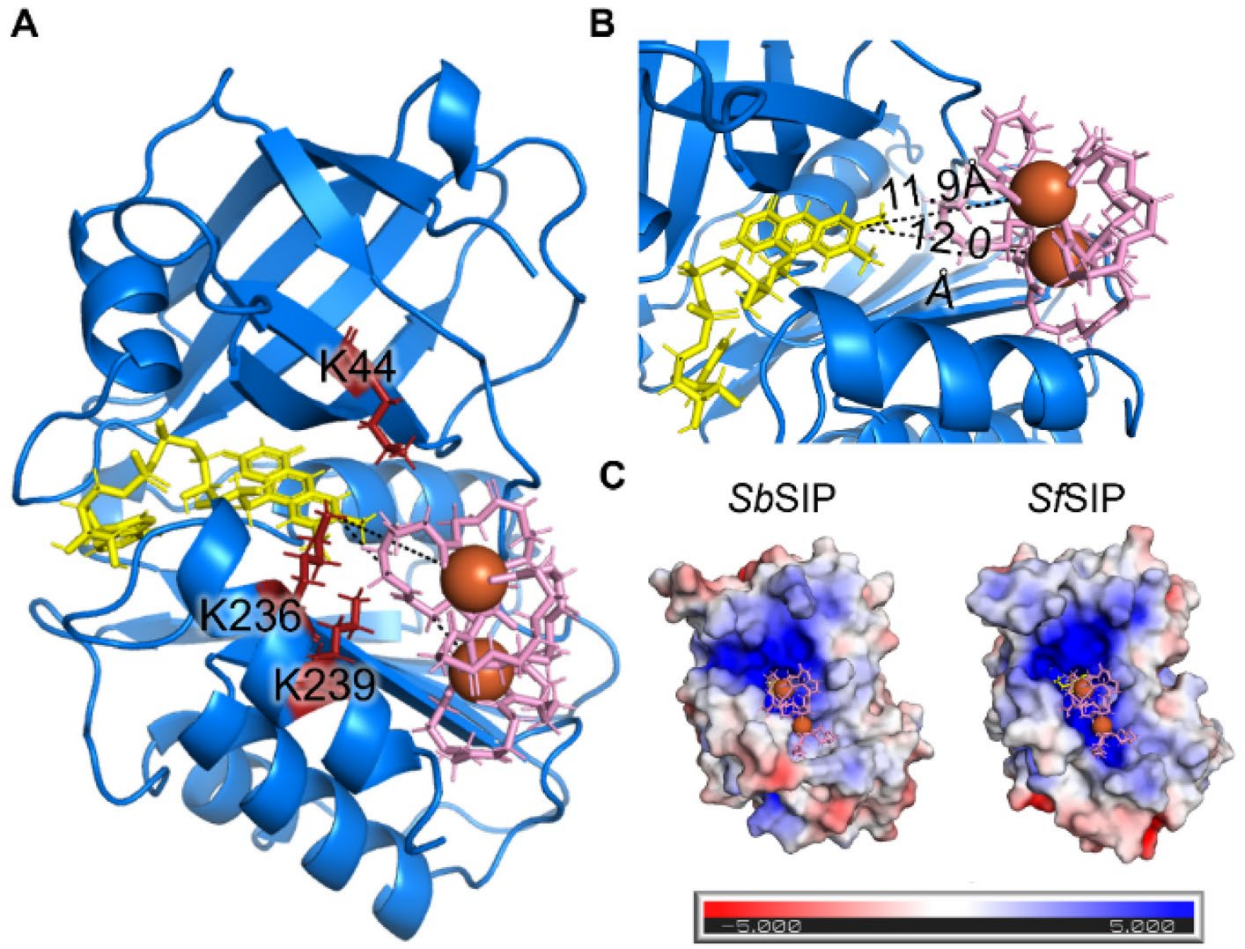
Representation of the binding conformations of Fe(III)–siderophores: **A** Docking of Fe(III)–bisucaberin (pink) with *Sb*SIP highlighting the lysine triad (red); **B** zoom of Fe(III)–bisucaberin binding pocket in *Sb*SIP highlighting the shortest distances between the isoalloxazine ring of the FAD cofactor and the two Fe(III) atoms of the Fe(III)–bisucaberin complex; **C** Electrostatic surface potential (− 5 to + 5 kT/e) of *Sb*SIP and *Sf*SIP and the respective Fe(III)–bisucaberin binding pockets

Ferric–siderophores undergo speciation according to pH and concentration ratio of ligand and iron [61].The most prevalent form at neutral pH of iron-chelated hydroxamate siderophores such as bisucaberin produced by *Shewanella* spp is a binuclear form with three ligands generally designated Fe_2_L_3_, and the structure of this species as well as that of the analogous siderophore alcaligin are available [61, 62]. Docking simulations of these siderophores with the structure of *Sb*SIP show that the binding pocket of Fe(III)–siderophores is the same where NADH and NADPH bind. The best docking solutions show distances between the isoalloxazine ring of *Sb*SIP and the Fe atoms in Fe(III)–bisucaberin of 11.9 and 12.0 Å (Fig. 8) and 11.0 and 14.2 Å for the case of Fe(III)–alcaligin (Fig. S8). In the case of *Sf*SIP, these distances are shorter with values of 9.0 and 12.5 for Fe(III)–bisucaberin and 9.4 and 13.0 Å for Fe(III)–alcaligin (Fig. S9), suggesting faster electron transfer [21].

To test the capability of *Sb*SIP to reduce ferric siderophores we made use of the decrease in optical absorption of the semiquinone state of the protein at 600 nm upon oxidation. This provides a clean readout of electron transfer that is testable by stopped-flow kinetic assays without interference from the UV–visible absorption bands of the siderophores. We did not have access to Fe(III)–alcaligin and therefore the experiments were performed using Fe(III)–bisucaberin and Fe(III)–putrebactin. Putrebactin is another hydroxamate siderophore produced by *Shewanella* spp, but in this case an experimentally determined structure of the physiologically relevant species is not available preventing the calculation of further docking conformations.

**Fig. 9:**
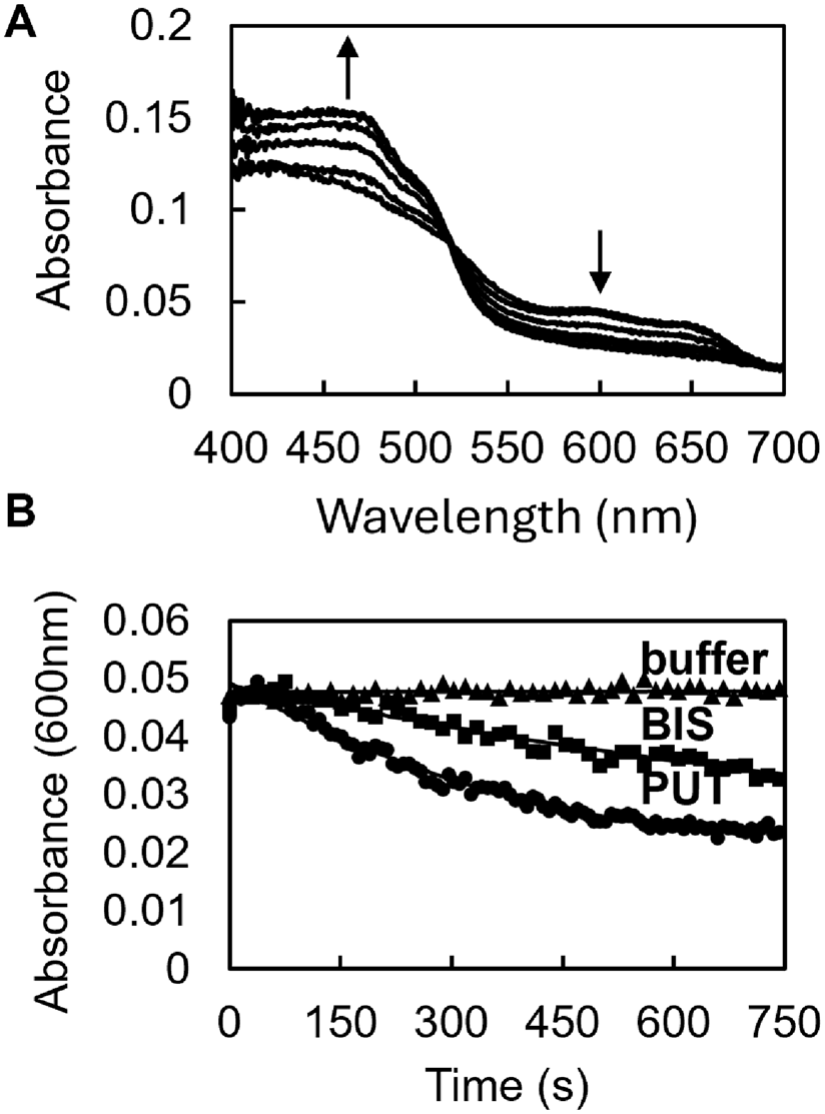
Fe(III)–siderophore reduction by *Sb*SIP: **A** absorption spectra changes after mixing *Sb*SIP_semi_ with Fe(III)–siderophore bisucaberin. Up arrow indicates the increase in absorbance at 470 nm and the down arrow indicates the decrease in the absorbance at 600 nm; **B** respective kinetic traces of *Sb*SIP at 600 nm, showing the oxidation of *Sb*SIP and concomitant reduction of bisucaberin (BIS) and putrebactin (PUT)

Upon mixing of *Sb*SIP_semi_ with ferric–siderophores bisucaberin (BIS) and putrebactin (PUT) absorption spectra changes showed an increase at 470 nm and a decrease at 600 nm (Fig. 9A). This is consistent with the transfer of one electron from the flavin in the semiquinone state to the Fe(III)–siderophore yielding fully oxidized *Sb*SIP and Fe(II). This result confirms the nature of *Sb*SIP as a NAD(P)H siderophore oxidoreductase. The rate constant for Fe(III)–siderophore reduction was determined to be 0.0014 ± 2 × 10^–4^ s^−1^ for BIS and 0.0031 ± 5 × 10^–5^ s^−1^ for PUT (Fig. 9B). The reduction rate constants for both Fe(III)–siderophores are of similar magnitude, but are almost one order of magnitude lower than those found for *Sf*SIP [21]. This difference cannot be attributed to different driving forces given the similar reduction potential and redox-Bohr effect for the transition between the oxidized and the semiquinone state of the two enzymes. On the other hand these results do match the docking predictions where shorter distances between FAD cofactor and Fe(III)–siderophores are observed for *Sf*SIP.

## Discussion

Here, we present the functional characterization and structure determination of a novel SIP from *S. bicestrii* (*Sb*SIP) and show that it is an NAD(P)H–siderophore oxidoreductase. The data also show that the redox-Bohr effect is likely to be a common functional feature of these enzymes [21]. The coupled transfer of electrons and protons in the physiological pH range is clearly advantageous for the activity of a ferric–siderophore reductase. It enhances the driving force for Fe(III)–siderophore reduction at high pH, where the solubility of Fe(III) is lower. Moreover, it ensures that iron reduction is accompanied by the release of a proton resulting in local pH reduction and in turn, enhanced Fe(II) solubility. Another possibility could be the protonation of the siderophore's hydroxamate groups, which would weaken the complex's affinity for iron, facilitating its dissociation.

The structure of *Sb*SIP presented an overall fold that is similar to previously characterized SIPs [12, 18, 21, 29]. However, the access pocket to the FAD cofactor is smaller than in the case of *Sf*SIP and likely sets the preference for NADH via steric clash with the 2′ phosphate group of NADPH. Miethke and co-workers proposed two different SIP subgroups based on the presence (subgroup I) or absence (subgroup II) of a longer C-terminal α-helical element [14]. Subgroup I would favor NADH binding, and subgroup II would favor NADPH binding. The SIPs from *E. coli* (YqjH) and from *T. fusca* (FscN) were shown to meet this criterion, belonging to subgroup II and binding NADPH-only. However, *Sf*SIP was shown to not meet the criterion, binding both NADH and NADPH with similar affinity, despite its classification as subgroup I Interestingly, *Sb*SIP that also belongs to subgroup I, shows a preference for NADH, suggesting that the extra C-terminal helix can indeed hinder access to the FAD pocket.

Our data also show that other factors modulate the activity SIPs toward NAD(P)H. Indeed, binding affinity of the putative physiological electron donors NADH and NADPH does not impact the rates of reduction of the enzymes, implying that electron transfer is the rate limiting step of the reaction. However, for Fe(III)–siderophore reduction, the rates are orders of magnitude slower for *Sb*SIP than for the previously characterized *Sf*SIP, despite similar redox potentials for the transition between the oxidized and semiquinone states. Given that the driving force is similar, the different rates may reflect different evolutionary pressures operating in the two organisms. The ecological settings of the two *Shewanella* sp. are quite distinct. *S. frigidimarina* was isolated from the Antarctic soil bed at very cold temperatures, whereas *S. bicestrii* was isolated from the much warmer condition of a human infection [33, 63]. The lower access to the FAD in *Sb*SIP may have been an evolutionary adaptation to slow down the rates of siderophore reduction at the higher temperature of the *S. bicestrii* habitat. This provides a kinetic control on the production of Fe(II) that may be essential to enable the metabolism to handle this essential but potentially toxic element while maintaining a similar thermodynamic driving force. Additionally, the lower reduction rates can also be a consequence of the fact that the genome of *S. bicestrii* codes for two ferric–siderophore reductases, *Sb*SIP and *Sb*FSR, whereas the genome of *S. frigidimarina* only codes for *Sf*SIP. Again, kinetic control of the relative activities of the two enzymes may be important in the metabolic regulation of iron availability, which operates on a much faster response time scale than transcriptional regulation. Nonetheless, it is also possible that the binding of siderophores is affected by temperature, either through modification of their affinity to *Sb*SIP or through the temperature dependence of speciation equilibria which changes iron availability. Two other relevant aspects of the distinct physiological context where *Sb*SIP and *Sf*SIP operate are: (i) the fact that very few studies exist regarding the exact siderophores, and corresponding Fe(III)–siderophore structures produced by the different species of *Shewanella*; (ii) and very few studies exist on which microorganisms form symbiotic relationships with the different *Shewanella* species. In this context, *Sb*SIP and *Sf*SIP may be tailored to target specific endogenously produced siderophores that are different between *S. frigidimarina* and *S. bicestrii*, and different from those used in this work. These enzymes may also be tailored to target particular xenosiderophores as observed in the case of *E. coli*’s native siderophore enterobactin. Most microorganisms do not synthesize this siderophore, but nonetheless express the Fe(III)–enterobactin esterase (Fes). This is also true for some *Shewanella* species, and it could provide a means to release iron when it is complexed with enterobactin, the strongest natural siderophore described to date [64].

The observation of *Sb*SIP reduction by NADH and by NADPH in the presence of the oxygen-scavenging system led us to revisit the reactivity of the previously characterized *Sf*SIP which also showed the same behavior (Fig. S10). Interestingly, these results suggest that oxygen is an efficient inhibitor of SIPs. This provides a mechanism to avoid the deleterious effect of free ferrous iron in the cell in the presence of oxygen, i.e., the production of reactive oxygen species through the Fenton reaction, by preventing the formation of ferrous iron at the source.

Finally, docking studies showed that siderophores bind *Sb*SIP and *Sf*SIP via the same binding pocket that binds NADH and NADPH, revealing a single hotspot that can be targeted to inhibit these enzymes. Given the structural diversity of siderophores, there are usually numerous Fe(III)–siderophore receptors at the surface of the cells to capture these essential molecules from the environment [65]. Also often, some siderophores require entire dedicated pathways for their utilization (e.g., enterobactin and pyoverdine) [11, 66]. However, other siderophores such as hydroxamates or products of enterobactin hydrolysis show that the extraction of the iron from the siderophore can be a metabolic choke point with few enzymes such as SIPs performing this task [12, 67]. The characterization of *Sb*SIP and the comparison with SfSIP reveal recurring structural and functional aspects of these enzymes that are now ripe for exploration and will likely be the make it or break it in the context of using these enzymes as targets to combat bacterial infections and antimicrobial resistance.

## Supplementary Information

Below is the link to the electronic supplementary material.

Supplementary file1 (DOCX 5228 KB)

## Data Availability

No datasets were generated or analyzed during the current study.
