# Supplementary material for: Flavin-containing siderophore-interacting protein of Shewanella putrefaciens DSM 9451 reveals common structural and functional aspects of ferric–siderophore reduction
Source: J Biol Inorg Chem. 2025 Mar 13;30(3):241–55. doi: 10.1007/s00775-025-02106-z (PMC11965169; doi:10.1007/s00775-025-02106-z)
Supplement: Supplementary file 1 — Supplementary file1 (DOCX 5228 KB) [file 775_2025_2106_MOESM1_ESM.docx]

**Supplementary Information for**

**Flavin-containing siderophore-interacting protein of *Shewanella putrefaciens* DSM 9451 reveals common structural and functional aspects of ferric-siderophore reduction**

Inês B. Trindade^1,2^, Bruno M. Fonseca^1^, Teresa Catarino^1,3^, Pedro M. Matias^1,4^, Elin Moe^1^, Ricardo O. Louro^1,*^

^1^ Instituto de Tecnologia Química e Biológica António Xavier da Universidade Nova de Lisboa, Avenida da República (EAN), 2780-157 Oeiras, Portugal

^2^ Current affiliation: Division of Biology and Biological Engineering, California Institute of Technology, Pasadena, CA 91125, USA

^3^ Departamento de Química, Faculdade de Ciências e Tecnologia, Universidade Nova de Lisboa, 2829-516, Caparica, Portugal

^4^ iBET – Instituto de Biologia Experimental e Tecnológica, Apartado 12, 2780-901 Oeiras, Portugal

* To whom correspondence should be addressed: [louro@itqb.unl.pt](mailto:louro@itqb.unl.pt)

| 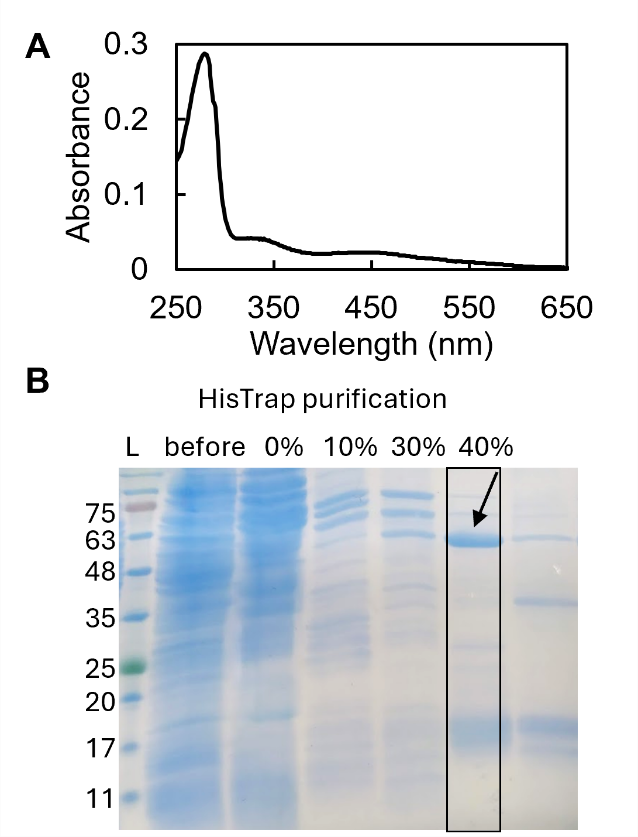 |
| --- |
| **Fig. S1** Production of *Sb*FSR. **A)** UV-visible profile of the fraction containing *Sb*FSR showing the typical bands at 340 nm and 451 nm. **B)** SDS-PAGE gel of *Sb*FSR purification. Boxes represent fractions used for further experiments and arrows show gel bands of proteins of interest. Percentages represent the amount of imidazole used out of a 500 mM stock solution. |
| \| **Table S1.** Top 20 hits DALI search regarding siderophore-interacting protein folds, highlighting in yellow unique SIP structures. \| \| \| \| \| \| \| \| \| --- \| --- \| --- \| --- \| --- \| --- \| --- \| --- \| \| **Job Query 8c4l-A** \| \| \| \| \| \| \| \| \| **No** \| **Chain** \| **Z** \| **rmsd** \| **lali** \| **nres** \| **%id** \| **PDB Description** \| \| **1** \| **8c4l-A** \| **44.5** \| **0** \| **243** \| **243** \| **100** \| **Siderophore-interacting protein from *Shewanella biscestrii*** \| \| **2** \| **8c4l-B** \| **42.1** \| **0.4** \| **241** \| **243** \| **100** \| **Siderophore-interacting protein from *Shewanella biscestrii*** \| \| **3** \| **2gpj-A** \| **40.4** \| **0.6** \| **242** \| **244** \| **82** \| **SIDEROPHORE-INTERACTING PROTEIN FROM *SHEWANELLA PUTREFACIENS* CN-32** \| \| **4** \| **7xlz-B** \| **33.7** \| **1.4** \| **233** \| **246** \| **45** \| **Siderophore-interacting protein from *Vibrio anguillarum*** \| \| **5** \| **7xlz-A** \| **32.8** \| **1.5** \| **231** \| **241** \| **46** \| **Siderophore-interacting protein from *Vibrio anguillarum*** \| \| **6** \| **6k2l-A** \| **29.6** \| **1.9** \| **235** \| **248** \| **36** \| **Siderophore-interacting protein SipS from *Aeromonas hydrophila*** \| \| **7** \| **6geh-A** \| **29.3** \| **2.2** \| **239** \| **256** \| **29** \| **Siderophore-interacting protein from *Shewanella frigidimarina*** \| \| **8** \| **7lrn-A** \| **26.7** \| **2.4** \| **234** \| **246** \| **25** \| **Siderophore Interacting Protein from *Acinetbacter baumannii*** \| \| **9** \| **4yhb-B** \| **26.6** \| **2.1** \| **228** \| **262** \| **29** \| **Siderophore utilization protein from *T. fusca*** \| \| **10** \| **7lrn-B** \| **26.5** \| **2.4** \| **234** \| **250** \| **25** \| **Siderophore Interacting Protein from Acinetbacter baumannii** \| \| **11** \| **4yhb-A** \| **26.3** \| **2.1** \| **227** \| **261** \| **29** \| **Siderophore utilization protein from *T. fusca*** \| \| **12** \| **6k2l-B** \| **25.6** \| **1.9** \| **212** \| **224** \| **38** \| **Siderophore-interacting protein SipS from *Aeromonas hydrophila*** \| \| **13** \| **6tek-A** \| **24.9** \| **2.5** \| **220** \| **230** \| **26** \| **Structure of siderophore interaction domain of IrtAB** \| \| **14** \| **6tek-B** \| **24.7** \| **2.5** \| **220** \| **230** \| **25** \| **Structure of siderophore interaction domain of IrtAB** \| \| **15** \| **4g1v-A** \| **20.2** \| **2.7** \| **215** \| **398** \| **16** \| **X-ray structure of yeast flavohemoglobin** \| \| **16** \| **4g1b-B** \| **20.1** \| **2.8** \| **216** \| **398** \| **16** \| **X-ray structure of yeast flavohemoglobin in complex with econazole** \| \| **17** \| **4g1b-D** \| **20** \| **2.7** \| **217** \| **398** \| **14** \| **X-ray structure of yeast flavohemoglobin in complex with econazole** \| \| **18** \| **4eh1-A** \| **19.9** \| **3** \| **212** \| **237** \| **14** \| **Flavohem-like-FAD/NAD Binding Domain of Nitric Oxide Dioxygenase from *Vibrio cholerae* O1** \| \| **19** \| **3ozv-A** \| **19.5** \| **2.9** \| **214** \| **403** \| **13** \| **Flavohemoglobin from R. eutrophus in complex with econazole** \| \| **20** \| **4uaj-A** \| **19.5** \| **3** \| **210** \| **280** \| **13** \| **NqrF in hexagonal space group** \| |

| 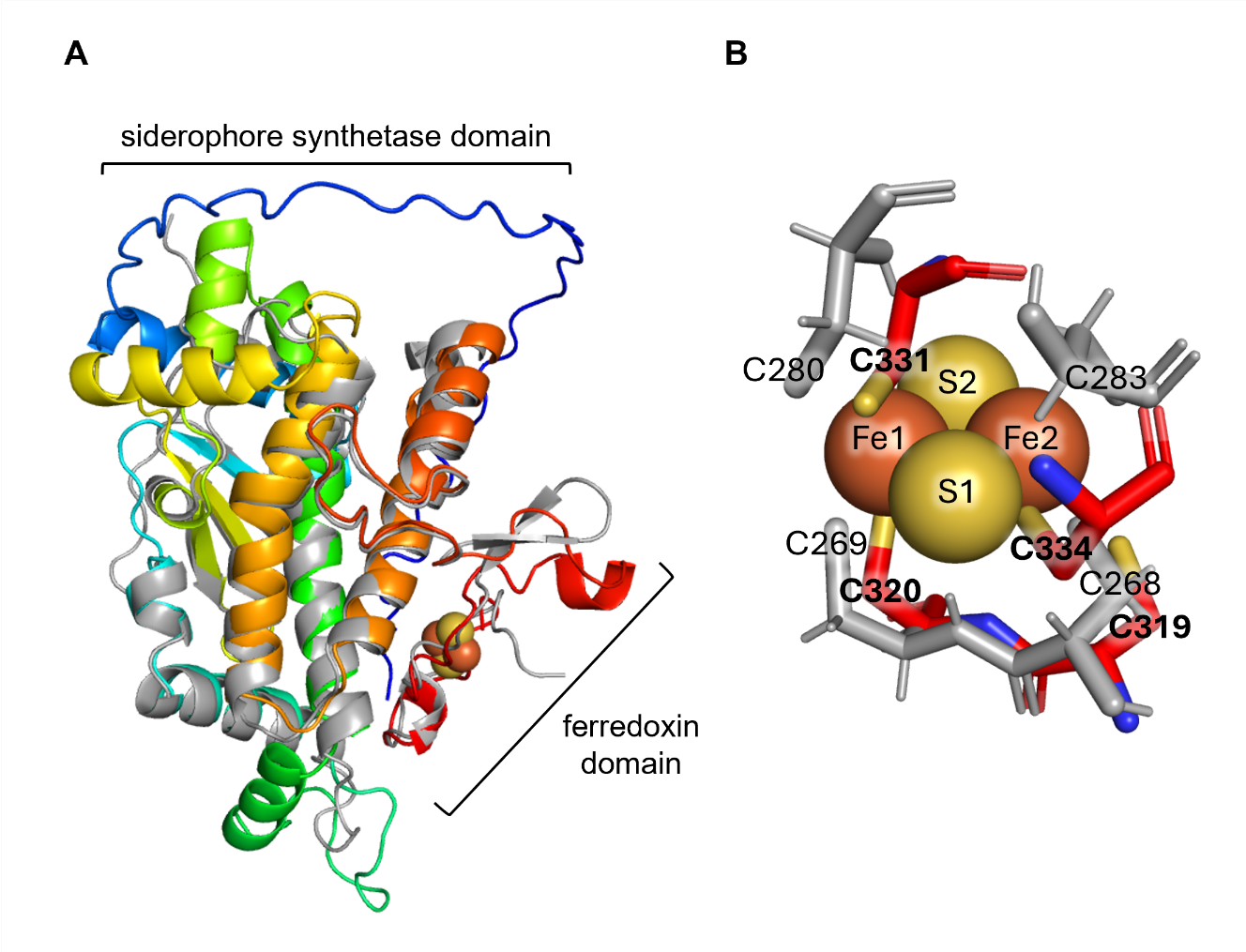 |
| --- |
| **Fig. S2** Structural comparison of *Sb*FSR with FhuF from *E. coli* **A)** *Sb*FSR structural model (blue to red from the N to the C terminal, AlphaFold model) vs the structure of FhuF (grey, PDB 7QP5) **B)** Close-up to the 2Fe-2S within the ferredoxin domain highlighting coordinating cysteines. |

| 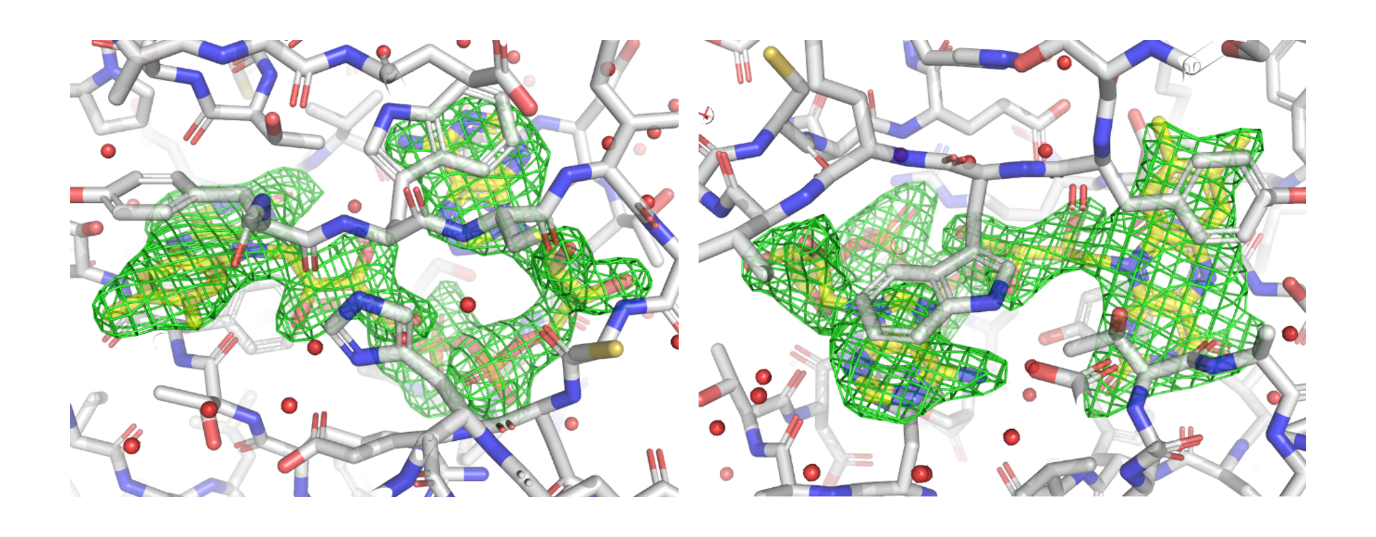 | |
| --- | --- |
| **Fig. S3** FAD omit map for molecule A (left) and B (right) of the crystallographic unit cell. | |
| \| **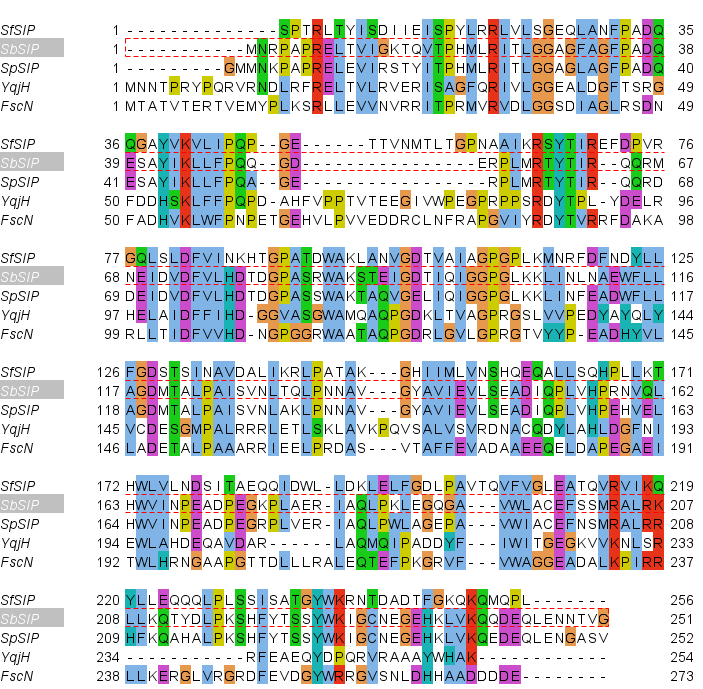** \| \| --- \| \| **Fig. S4** Multiple sequence alignment of SIPs produced using Jalview with default color scheme used for alignments in Clustal X. \|  \| **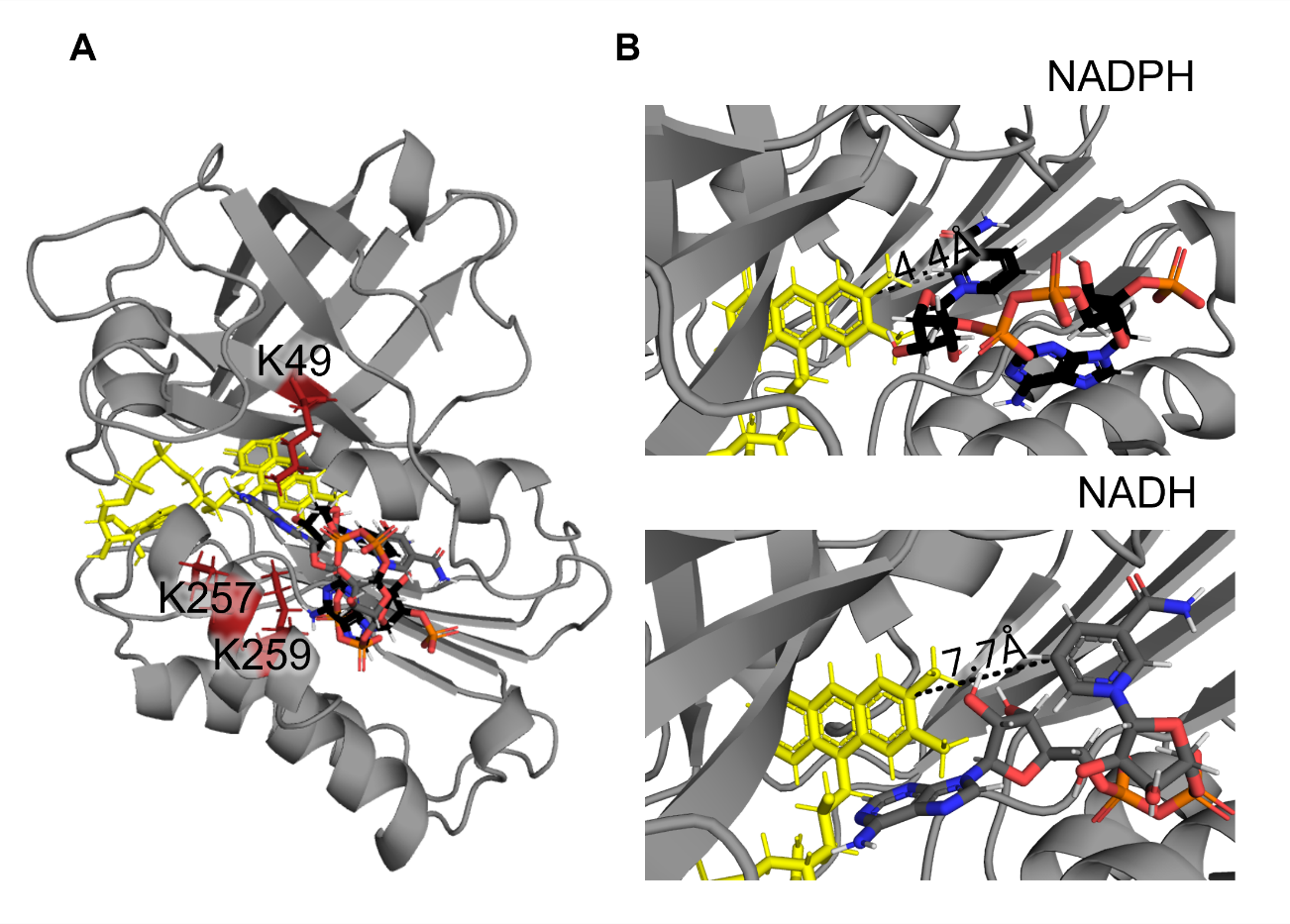** \| \| --- \| \| **Fig. S5** Representation of the binding conformations of NADPH and NADH to *Sf*SIP calculated using HADDOCK. A) The binding of NADH (gray) and NADPH (black) occurs in the same region of the isoalloxazine ring of the FAD cofactor (yellow) which is surrounded by the lysine triad (red). (B) zoom of NADPH (top) and NADH (bottom) binding pockets highlighting the shortest distances found between the FAD cofactor and NADPH and NADH respectively. \| | |
|  | |
|  | |
| \| 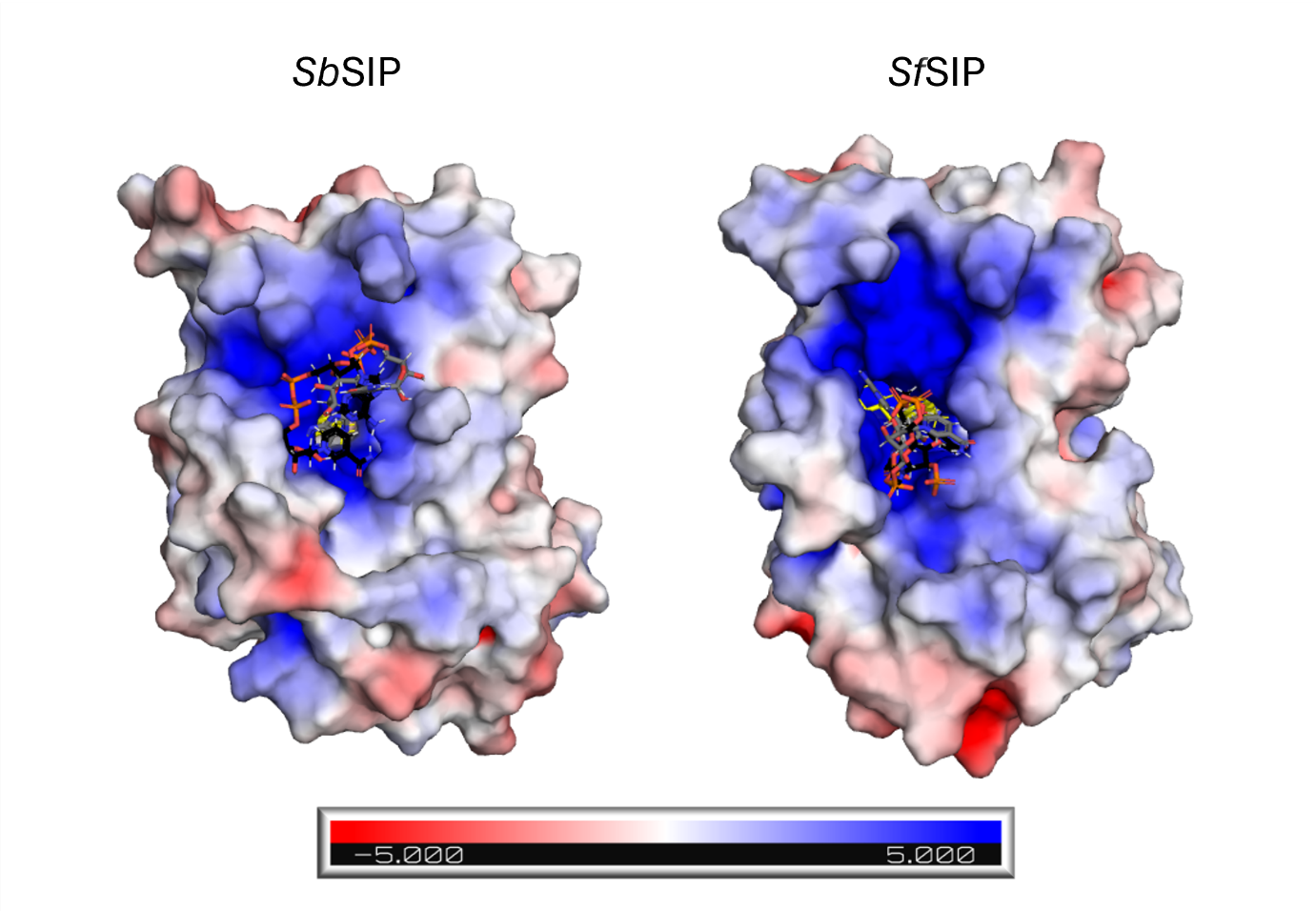 \| \| --- \| \| **Fig. S6.** Electrostatic surface potential (−5 to +5 kT/e) of *Sb*SIP and *Sf*SIP and respective NADH (gray) and NADPH (black) binding pockets. \|  \| 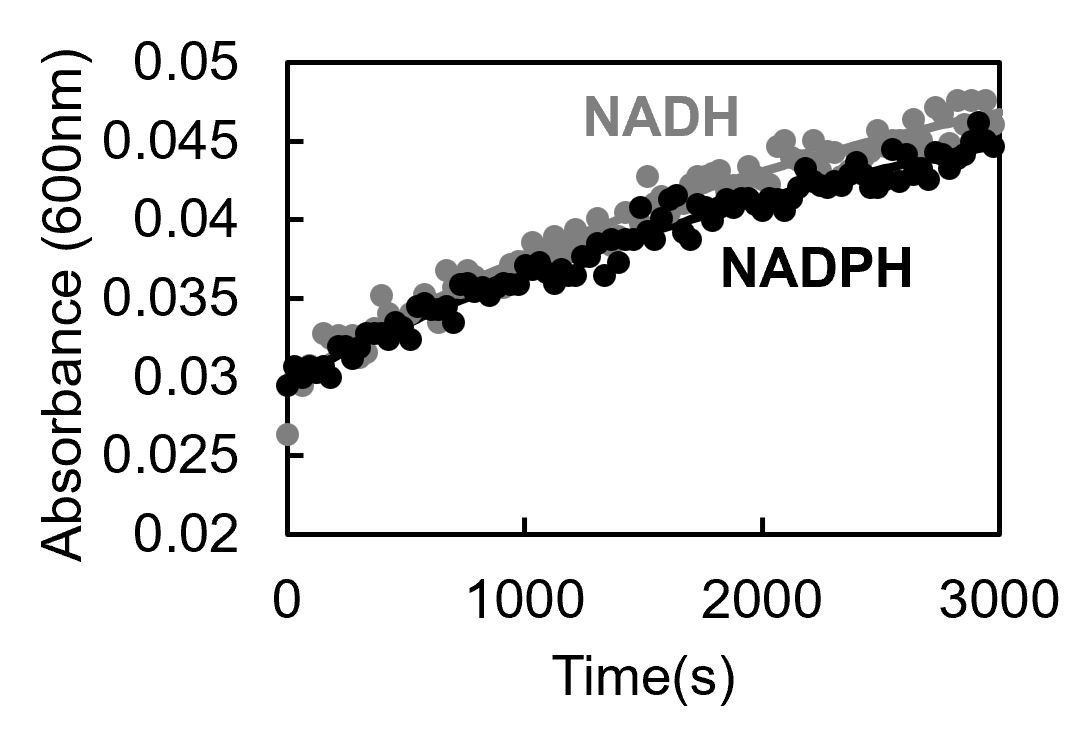 \| \| --- \| \| **Fig. S7** Kinetic trace at 600nm of the changes observed after mixing *Sb*SIP with NADH and NADPH. Data for NADH is reported in gray and for NADPH in black. \|  \| **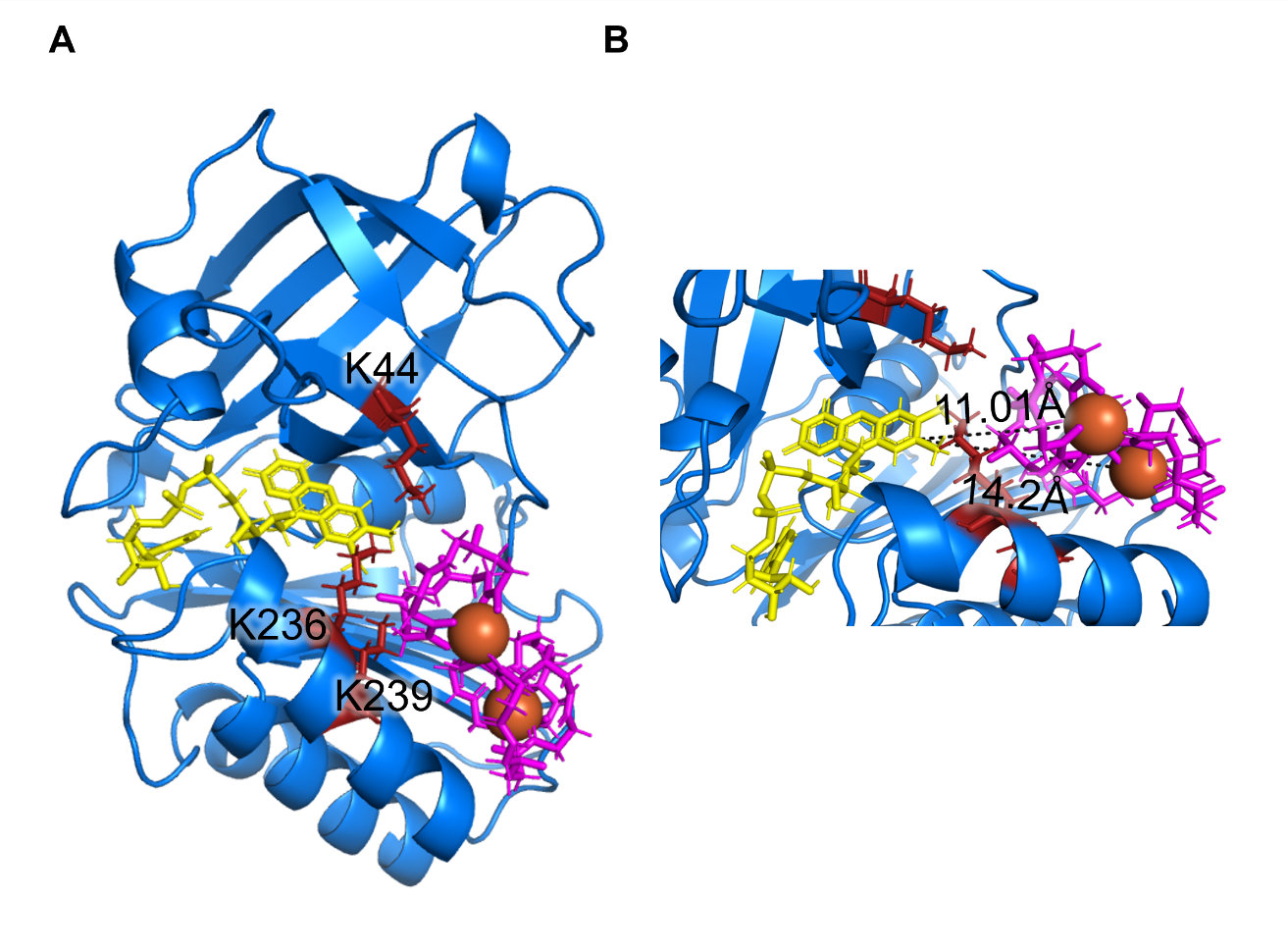** \| \| --- \| \| **Fig. S8** Representation of the binding conformations of Fe(III)-alcaligin with *Sb*SIP calculated using HADDOCK: A) Docking of Fe(III)-alcaligin (magenta) with *Sb*SIP highlighting the lysine triad (red); B) Zoom of Fe(III)-alcaligin binding pocket highlighting the shortest distances between the isoalloxazine ring of the FAD cofactor and the two Fe(III) atoms of the Fe(III)-alcaligin complex. \|  \| **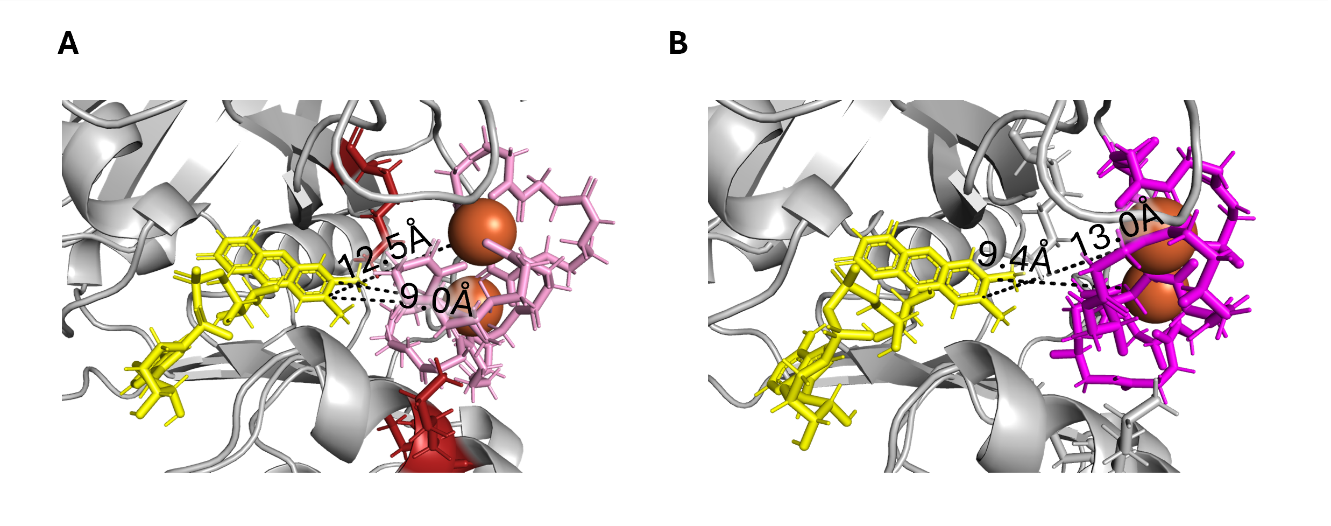** \| \| --- \| \| **Fig. S9**. Representation of the binding conformations of Fe(III)-siderophores with *Sf*SIP calculated using HADDOCK: A) Docking of Fe(III)-bisucaberin (pink) and B) Fe(III)-alcaligin (magenta) with *Sf*SIP highlighting the lysine triad (red) and the shortest distances between the isoalloxazine ring of the FAD cofactor and the Fe(III) atoms of the Fe(III)-siderophore complexes. \|  \| 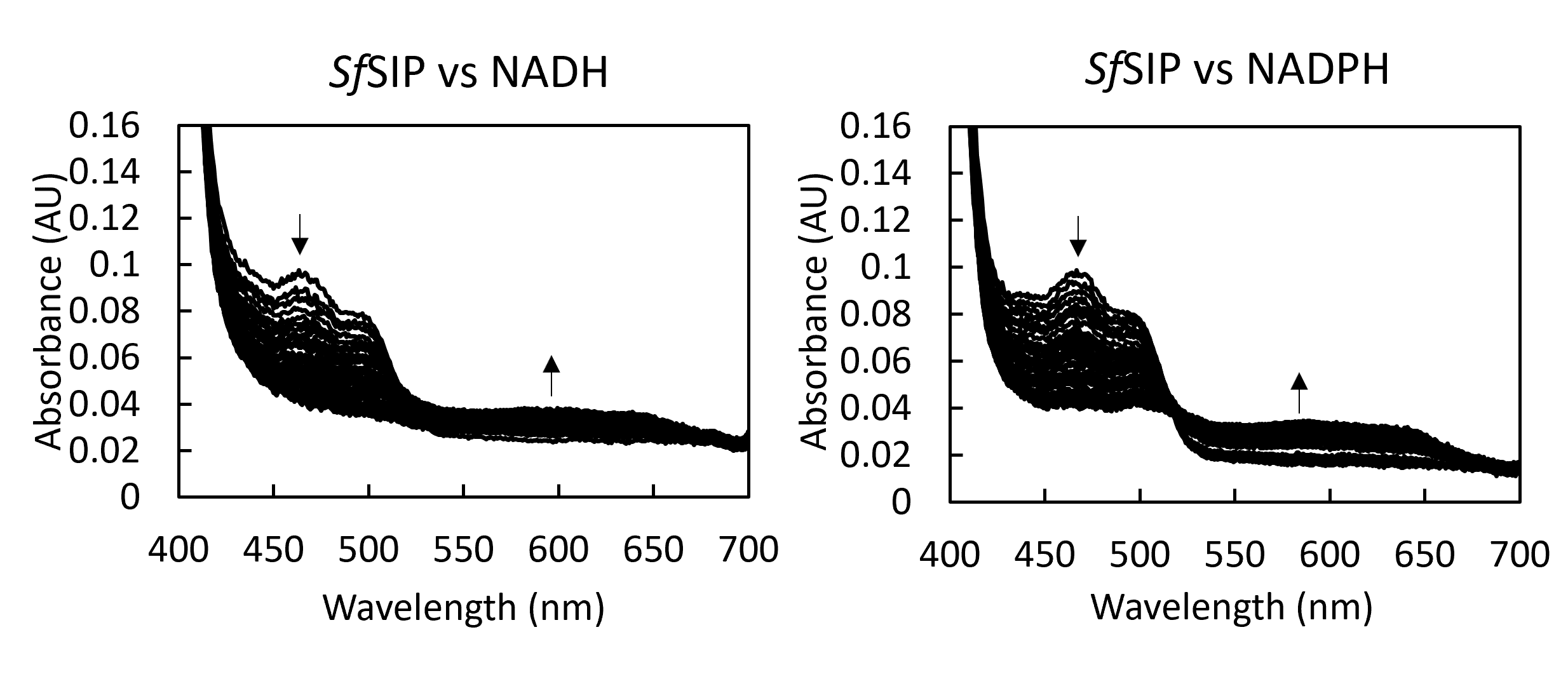 \| \| --- \| \| **Fig. S10** Reduction of *Sf*SIP using NADH and NADPH in the presence of an oxygen-scavenging system. **A)** Representative UV-visible spectral changes upon mixing SfSIP with NADH and **B)** NADPH. Arrows indicate the decrease at 470 nm and increase at 600 nm indicating the formation of the semiquinone state. \| | |
|  |  |
